# Novel Imidazole and Methoxybenzylamine Growth Inhibitors Affecting *Salmonella* Cell Envelope Integrity and its Persistence in Chickens

**DOI:** 10.1038/s41598-018-31249-0

**Published:** 2018-09-06

**Authors:** Loïc Deblais, Yosra A. Helmy, Dipak Kathayat, Huang-chi Huang, Sally A. Miller, Gireesh Rajashekara

**Affiliations:** 1 Food Animal Health Research Program, Department of Veterinary Preventive Medicine, The Ohio State University, OARDC, Wooster, OH USA; 2 Department of Plant Pathology, The Ohio State University, OARDC, Wooster, OH USA

**Keywords:** Bioinformatics, High-throughput screening

## Abstract

The control of *Salmonella* from farm to fork is challenging due to the emergence of antimicrobial-resistant isolates and the limited effects of current control methods. Advanced chemical technologies have made accessible a wide range of uncharacterized small molecules (SMs) with encouraging chemical properties for antimicrobial treatment. Of the 4,182 SMs screened *in vitro*, four cidal SMs were effective at 10 µM and higher against several serotypes, antibiotic-resistant, and biofilm embedded *Salmonella enterica* subsp. *enterica* serotype Typhimurium by altering cell membrane integrity. The four SMs displayed synergistic effects with ciprofloxacin, meropenem and cefeprime against *Salmonella*. Further, the SMs were not pernicious to most eukaryotic cells at 200 μM and cleared internalized *Salmonella* in infected Caco-2, HD11, and THP-1 cells at 6.25 µM and higher. The SMs also increased the longevity of *Salmonella*-infected *Galleria mellonella* larvae and reduced the population of internalized *Salmonella* Typhimurium. Two of the SMs (SM4 and SM5) also reduced *S*. Typhimurium load in infected chicken ceca as well as its systemic translocation into other tissues, with minimal impact on the cecal microbiota. This study demonstrated that SMs are a viable source of potential antimicrobials applicable in food animal production against *Salmonella*.

## Introduction

Non-typhoidal *Salmonella* are common causes of human food poisoning worldwide (https://www.ers.usda.gov/data-products/cost-estimates-of-foodborne-illnesses/). Contaminated poultry products are the most common sources of *Salmonella* infections in humans^1–3^. *Salmonella* can colonize the gastrointestinal track of chickens at high density within a few days after infection and without causing any clinical symptoms, which significantly increases the risk of post-slaughter contamination of the products^3^. For example, a recent study showed that approximately 11% of the chicken breasts purchased in U.S. retailers were contaminated with *Salmonella*^4^. In some cases, a prolonged infection of chickens can lead to bacteremia followed by the colonization of internal organs such as spleen, liver, and ovaries^3^. Infected chickens can rapidly disseminate *Salmonella* through the whole flock via persistent shedding of the pathogen in the feces or through vertical transfer to the next generation via eggs^5^. Therefore, an early infection can results in contamination of the farm environment and a high morbidity^5,6^. Despite detailed knowledge about *Salmonella* infection in chickens, the salmonellosis incidence rate in human remains the same over the past 20 years^7^. It was estimated that the economical and public health burden of *Salmonella* is between $2.3 and 11.3 billion annually in the U.S., and approximately up to 30.3% of this cost is due to poultry-associated *Salmonella* infections^8,9^. *Salmonella* can be detected in various poultry-associated products, including pasteurized eggs (14.6%), ground turkey (49.9%), and ground chicken meat (44.6%) in the U.S.^10^. Further, over 70 backyard poultry-associated salmonellosis outbreaks have been reported in the U.S. since 2000, causing 4,794 illnesses, about 894 hospitalizations, and seven deaths^11^.

Pre-harvest control methods (competitive exclusion, vaccination, and antimicrobial supplementation in water/feed) are available to reduce on-farm and post-slaughter contaminations of the carcasses; however their effects are limited or easily overcome by *Salmonella* due to constant adaptation of *Salmonella* to these management strategies^5^. For example, approximately 100,000 salmonellosis cases are caused by multi-drug resistant *Salmonella* strains annually in the U.S.^12^. Further, *Salmonella* isolates resistant to two important groups of antibiotics (cephalosporins and fluoroquinolones) that are extensively used against *Salmonella* in food animals and humans have been reported^12,13^. Therefore, the development of new antimicrobials effective against *Salmonella* and with novel modes of action is needed to counter the *Salmonella* burden and improve public health^14^.

Over the past decade, pharmaceutical companies have developed thousands of new generation small molecules (SMs). Some of these SMs have been shown to be effective against multi-drug resistant pathogens such as *Staphylococcus*, *Burkholderia*, *Pseudomonas*, and *Candida*, where conventional antibiotics failed^15–18^. These SMs present characteristic physico-chemical properties designed to enhance their antimicrobial efficacy as well as their industrial applications. For example, their low molecular weight and high hydrophilicity enhance their absorption and permeation throughout host and pathogen barriers^19–21^. Further, the structural novelty of these SMs could be associated with novel antimicrobial modes of action. Therefore, new generation SMs might represent a source of novel antimicrobials to control foodborne pathogens such as *Salmonella*.

The objective of this study was to identify novel growth inhibitors small molecules effective against *Salmonella* in chickens. After screening a library of 4,182 SMs, our study identified two novel potent SMs effective at low concentration against various serotypes, antibiotic-resistant, and biofilm embedded *Salmonella*. These SMs possessed low toxicity to eukaryotic cells and were effective in reducing *Salmonella* in *Galleria mellonella* wax moth larvae and in chickens with minimal impact on the chicken cecal microbiota. Further, cytological profiling revealed that these SMs function by altering *Salmonella* cell membrane integrity.

## Results

### Nineteen SMs completely inhibited *Salmonella enterica* subsp. *enterica* serotype Typhimurium growth at 200 µM

A high-throughput screening of 4,182 SMs was conducted using 200 µM of SMs against *S*. Typhimurium LT2 wild-type (WT) strain in a 96-well plate format in order to identify novel SM growth inhibitors. A total of 128 SMs inhibited *S*. Typhimurium growth between 20% to 100% when *Salmonella* was grown in minimal nutrient conditions (M9 medium) for 12 hrs. Among the 128 SMs, 10 SMs were bacteriostatic (no increase in optical density (OD) at 600 nm but growth recovered on agar medium after 12 hrs of treatment) and nine had a bactericidal effect (no increase in OD at 600 nm and no growth on agar medium after 12 hrs of treatment) at 200 µM.

A dose-response assay was performed with the 19 SMs that completely inhibited *S*. Typhimurium growth in the primary screening. One SM (SM4) had a minimal bactericidal concentration (MBC) of 10 µM; two SMs (SM3 and SM5) had a MBC of 25 µM; two SMs (SM1 and SM7) had a MBC of 50 µM; two SMs had a MBC of 100 µM (SM8 and SM2); four SMs had a MBC of 200 µM (SM6 and SM9); six SMs had a MBC of 400 µM (SM10–15); and four SMs (SM16-SM19) had a minimal inhibitory concentration (MIC) of 200 µM but their MBC was not determined (>400 µM). Details concerning the 19 SMs chemical properties are displayed in Table S1.

### Five SMs completely inhibited the growth of several *Salmonella* serotypes at low concentration

A spectrum of activity was assessed at 200 µM against eight *Salmonella* serotypes, commonly implicated in foodborne salmonellosis, using the 128 SMs that inhibited at least 20% of *S*. Typhimurium growth (see Supplementary Fig. S1 & Supplementary Table S2). *S*. Typhimurium and *S*. Newport were the two serotypes with the highest number hits (SMs with a bactericidal or bacteriostatic effect) at 200 µM (n = 19 and 18, respectively), while *S*. Anatum and *S*. Heidelberg had the lowest number of hits (n = 8 for both). Among the 19 hits identified with *S*. Typhimurium (Supplementary Table S1), bactericidal SMs (SM1 to SM9) had a broader spectrum of activity than bacteriostatic SMs (SM10 to SM19). Five SMs (SM1 to SM5) were cidal to all nine *Salmonella* serotypes at 200 µM in M9 broth and displayed similar MBC values against *S*. Typhimurium in LB medium.

Based on the spectrum of activity and the dose-response assay, five SMs (SM1-SM5) were selected for a dose-response assay on all *Salmonella* serotypes tested above (Table 1). Among the nine serotypes, the MBCs ranged between 50 µM and 100 µM for SM1; between 100 µM and 200 µM for SM2; between 25 µM and 50 µM for SM3; between 10 µM and 25 µM for SM4; and between 25 µM and 100 µM for SM5 in M9 broth.

**Table 1:** Antimicrobial efficacy of the selected five small molecules (SMs) on different *Salmonella* *enterica* serotypes.

*Salmonella* serovars	MBC (μM)				
	SM1	SM2	SM3	SM4	SM5
Typhimurium	50	100	25	10	25
Albany	100	200	25	10	100
Anatum	100	200	25	10	50
Braenderup	100	200	25	10	50
Enteritidis	100	200	25	10	50
Heidelberg	100	200	50	25	50
Javiana	100	200	25	10	50
Newport	100	200	50	25	50
Saint-Paul	100	200	50	10	50
Muenchen	100	ND	50	25	50

ND: not determined; MBC: minimal bactericidal concentration.

### No resistance from *S*. Typhimurium was detected with four SMs

When tested with a lethal dose (2X MBC) on a solid medium or with repeated exposure to a sub-lethal dose (0.75X MBC) in a liquid medium, no resistance was observed with SM1, SM3, SM4, and SM5. *S*. Typhimurium developed resistance to SM2 following a single exposure at a lethal dose (2X MBC) in solid M9 medium, and also during repeated exposures (15 passages of 12 hrs each) to a sub-lethal dose (0.75X MBC) in liquid M9 medium, at 37 °C. The resistant bacteria were able to grow in M9 broth with 400 µM SM2 (4X MBC). Nevertheless, these resistant bacteria displayed similar sensitivity to the other four SMs, suggesting that SM2 probably has a different target in *Salmonella* than the other four SMs. Only SM1, SM3, SM4, and SM5 were selected for all the experiments described bellow.

### The four selected SMs were effective on antibiotic-resistant *Salmonella* as well as other poultry-associated pathogenic bacteria

**Figure 1:**
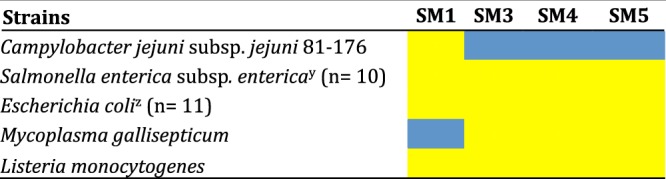
Activity spectrum of the selected four small molecule (SMs) at 200 μM on several foodborne and avian pathogens. Yellow cells: cidal effect; blue cells: bacterial growth observed; ^x^*Salmonella* *enterica* serotypes (Typhimurium, Albany, Anatum, Braenderup, Enteritidis, Heidelberg, Javiana, Muenchen, Newport, and Saint-Paul); ^z^avian pathogenic *E. coli* O1, O2, O8, O15, O18, O35, O78, O109, and O115 serotypes, and two enterohemorrhagic *E. coli* O157:H7 strains; n: number of strains/serotypes cluster within the same bacterial species.

The four selected SMs were bactericidal at 200 µM against six *S*. Typhimurium strains resistant to sulfamethoxazole, streptomycin, oxytetracycline, ampicillin, ciprofloxacin, and/or trimethoprim-sulphamethoxazole (Supplementary Table S2). The four SMs were also effective against several avian pathogenic *Escherichia coli* serotypes (O1, O2, O8, O15, O18, O35, O78, O109, and O115) at 100 µM, and enterohemorrhagic *E. coli* (EHEC O157:H7) and *Listeria monocytogenes* strains at 200 µM. SM1 was also lethal to *Campylobacter jejuni* 81–176 at 200 µM, while SM3, SM4, and SM5 were lethal to avian *Mycoplasma gallisepticum* at 100 µM (Fig. 1; unpublished data).

### The four selected SMs enhanced the antimicrobial efficacy of antibiotics

The potentiation effect of the four SMs was tested with six antibiotics (ciprofloxacin, nalidixic acid, meropenem, cefeprime, cefotaxime, and erythromycin) commonly used against *Salmonella* in poultry and humans, using a checkerboard assay. Out of the six antibiotics tested, three (ciprofloxacin, cefeprime, and meropenem) had a synergistic or additive effect with at least one of the four SMs tested (Table 2). SM1 displayed the best potentiation results, followed by SM3, SM4, and SM5. All SMs reduced ciprofloxacin MBC by at least 15.6-fold; SM1, SM3, and SM4 reduced the cefeprime MBC by at least 5-fold; and SM1 and SM3 reduced the meropenem MBC by 5 and 2.5-fold, respectively, when a sub-lethal concentration of SM was used.

**Table 2:** Combination effects of small molecules (SMs) and antibiotics on *Salmonella* *enterica* subsp. *enterica* serotype Typhimurium.

Antibiotics	AB MBC_alone_ (μg/ml)	SM1 (MBC_alone_ = 50 μM)		SM3 (MBC_alone_ = 25 μM)		SM4 (MBC_alone_ = 10 μM)		SM5 (MBC_alone_ = 25 μM)	
		AB MBC_combined_	SM1 MBC_combined_	AB MBC_combined_	SM3 MBC_combined_	AB MBC_combined_	SM4 MBC_combined_	AB MBC_combined_	SM5 MBC_combined_
Ciprofloxacin	0.0625	0.001^b^	40^b^	0.004^b^	15^b^	0.004^b^	7.5^b^	0.004^b^	20^b^
		0.004^a^	20^a^	0.006^a^	10^a^	0.006	10^c^		
		0.006^a^	5^a^	0.008^a^	5^a^				
Erythromycin	200	200^c^	50	200^c^	25^c^	200^c^	10^c^	200^c^	25^c^
Cefotaxime	3.2	3.2^c^	50	3.2^c^	25^c^	3.2^c^	10^c^	3.2^c^	25^c^
Nalidixic acid	16	4.8^c^	40	16^c^	25^c^	16^c^	10^c^	16^c^	25^c^
		8^c^	30						
Cefeprime	2	0.2^a^	20^a^	0.4^b^	20^b^	0.4^c^	10^c^	2^c^	25^c^
		1.2^b^	10^b^	0.8^b^	15^b^	1.6^b^	1.25^b^		
		1.6^b^	5^b^	1.2^b^	2.5^b^				
Meropenem	0.2	0.04^b^	20^b^	0.08^c^	20^c^	0.2^c^	10^c^	0.2^c^	25^c^
		0.08^b^	10^b^	0.16^c^	15 ^c^				
		0.16^b^	5^b^						

^a^Synergetic effect (FBC ≤ 0.5; reduction in MBCs superior to 75% for both antibiotic and SM). ^b^Additive effect (FBC > 0.5 and ≤ 1.0; percentage reduction in MBCs between 50% and 75% for both antibiotic and SM). ^c^Indifferent (FBC > 1.0 and ≤2.0; reduction in MBCs inferior to 50% for both antibiotic and SM). Antibiotic values in μg/ml. SM values in μM. MBC: minimal bactericidal concentration; AB: antibiotic; FBC: fractional bactericidal concentration.

### Selected four SMs were effective on biofilm embedded *Salmonella*

The antimicrobial efficacy of the four compounds (SM1, SM3-SM5) was tested on biofilm embedded *S*. Typhimurium using the MBEC (minimal biofilm eradication concentration) high-throughput assay^22^. After 18 hrs incubation of *Salmonella* with a SM concentration ranging between 0.2X MBC to 4X MBC, biofilm embedded *Salmonella* treated with SM5 displayed similar MBC value (25 µM) as in the dose-response assay performed with planktonic cells. On the other hand, biofilm embedded bacteria treated with SM1, SM3, and SM4 had a reduction in MBC values compared to the dose-response assay performed on planktonic cells. The SM1, SM3, and SM4 were cidal to biofilm embedded bacteria at 0.8X MBC (40 µM), 0.6X MBC (15 µM), and 0.4X MBC (4 µM), respectively compared to the dose-response assay performed in parallel with planktonic *Salmonella*. The increased antimicrobial susceptibility observed with the biofilm embedded *Salmonella* towards SM1, SM3, and SM4 suggest that biofilm embedded bacteria might display significant biological modification enhancing the antimicrobial activity of some of the SMs.

### Structure-activity relationship analysis

**Figure 2:**
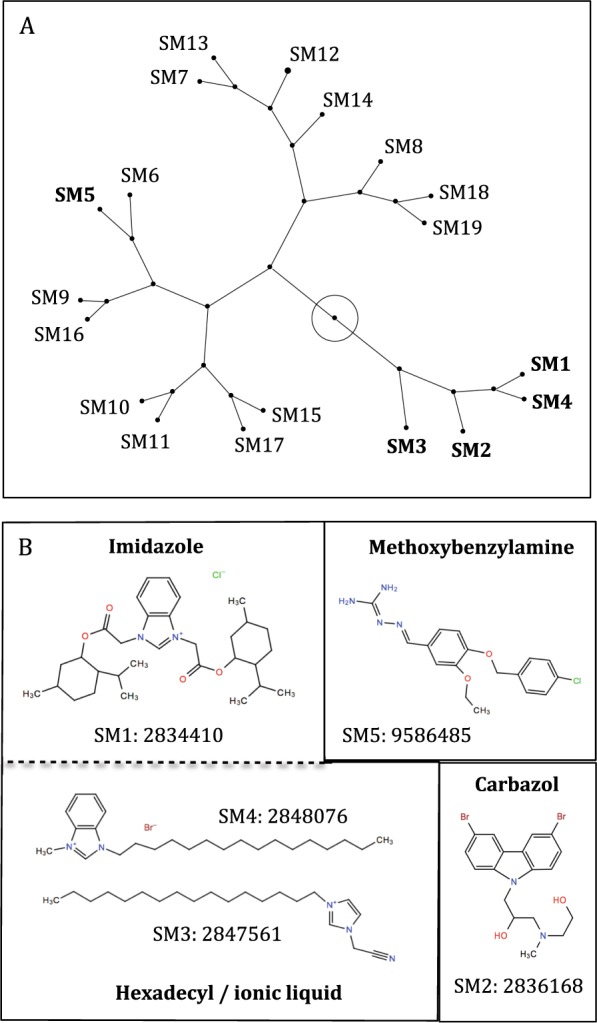
Structural analysis of the 19 small molecules (SMs) that completely inhibited *Salomella* *enterica* subsp. *enterica* serotype Typhimurium growth. (**A**) Constellation plot of the selected 19 SMs based on their two-dimensional structural similarities. In bold: SMs effective against nine *Salmonella* serovars. The root of the tree is represented by the circle within the plot. (**B**) Skeletal chemical formula of the selected five SMs. SMs were clustered based on the main chemical group with estimated antimicrobial properties. Serial number: PubChem ID.

Two-dimensional structural analysis of the 19 SMs inhibiting *S*. Typhimurium growth separated the SMs into three clusters based on a 2D Tanimoto scoring method (n = 4, 8, and 7; Fig. 2A). The two large clusters (n = 7 and 8) had a homogenous distribution of bacteriostatic and bactericidal SMs, while the small cluster (n = 4) was only composed of the SMs cidal against the nine *Salmonella* serovars at 200 µM (SM1, SM2, SM3, and SM4). SM5 was in the cluster of eight SMs. These results suggest that SM1, SM2, SM3, and SM4 have a common 2D structure that might explain the scope of their antimicrobial activity. SM1, SM3, and SM4 are potential ionic liquids composed of an imidazole group, SM2 is composed of a carbazol group, and SM5 is composed of a benzylamine group (Fig. 2B).

### SMs exhibited antimicrobial activity by affecting cell membrane integrity of *S*. Typhimurium

**Figure 3:**
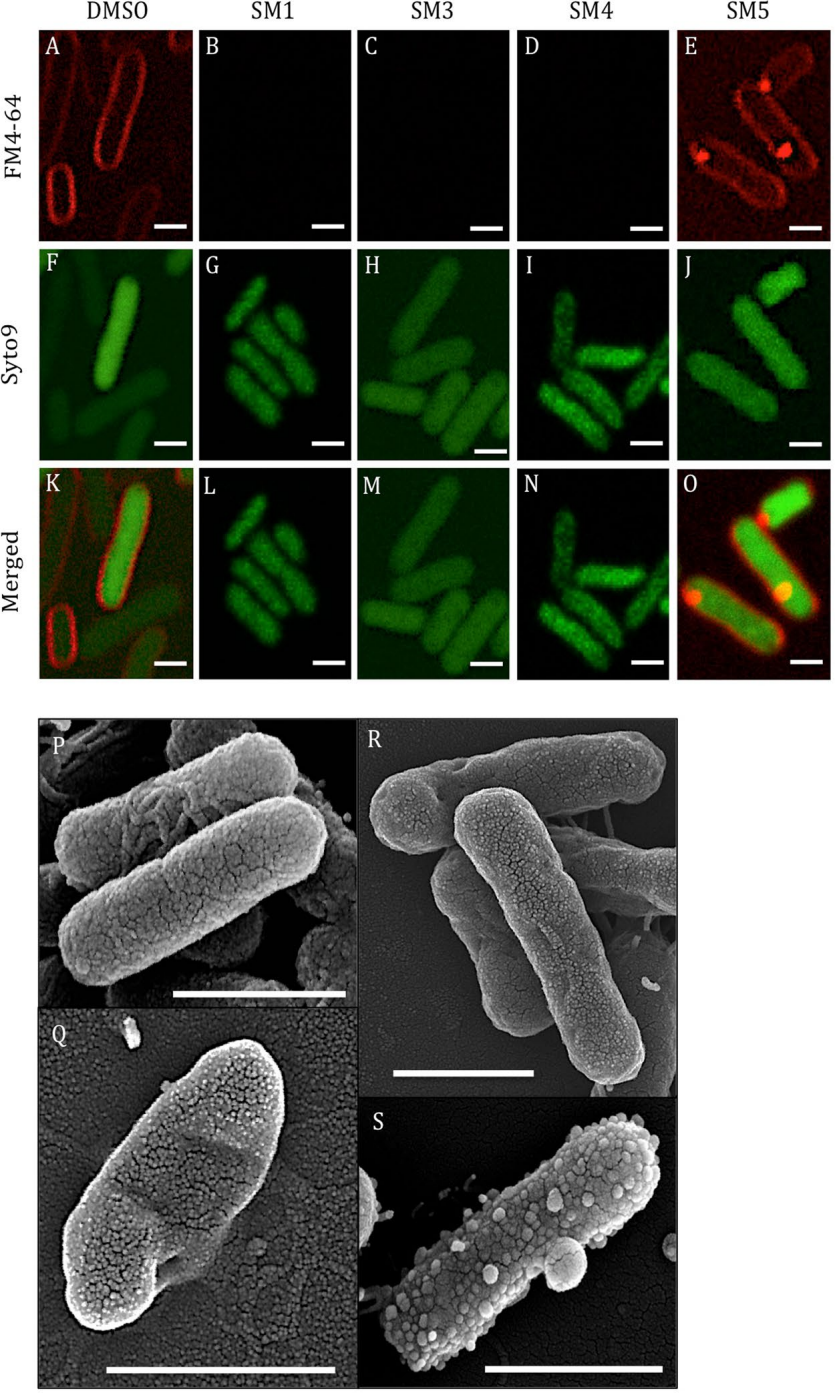
Confocal and scanning electron microscopy (SEM) analyses of *Salmonella* *enterica* subsp. *enterica* serotype Typhimurium after challenge with five times the minimal bactericidal concentraiton of small molecules (5X MBC of SMs) for 3 hrs. (**A**–**O**) Confocal microscopy: (**A**–**E**) *S*. Typhimurium cell membrane stained with FM4-64; (**F**–**J**) *S*. Typhimurium nucleic acids stained with SYTO9; (**K**–**O**) Merged pictures of the FM4-64 and SYTO9 staining. (**P**–**S**) SEM: (**P**) 1% dimethyl sulfoxide (DMSO) treated *Salmonella*; (**Q**) SM1 treated *Salmonella*; (**R**) SM4 treated *Salmonella*; (**S**) SM5 treated *Salmonella*; Bar: 1 μm.

Confocal microscopy analysis of *S*. Typhimurium challenged individually with a lethal dose of each of the four SMs revealed an alteration of the membrane phenotype when stained with FM4–64 compared to the 2% DMSO treated control (Fig. 3). No signal was detected from the FM4–64 staining when bacteria were treated with SM1, SM3, and SM4 (Fig. 3B–D) compared to the DMSO control (Fig. 3A). On the other hand, bacteria treated with SM5 displayed a stained cell membrane; however, a bright red spot was detected within every bacterium (Fig. 3E). No distinct modification of the phenotype was observed in bacteria treated with any of the four SMs after staining with the nucleic acid stain SYTO9 (Fig. 3G–J) compared to the DMSO control (Fig. 3F).

To further support the observation obtained with confocal microscopy, the same samples were analyzed using scanning electron microscopy (SEM; Fig. 3P–S). As expected, SM1-, SM4-, and SM5-treated cells displayed significant alterations of the cell surface (Fig. 3Q,R and S, respectively) compared to the 2% DMSO control (Fig. 3P) consistent with the confocal microscopy results (Fig. 3B–E). Further, the FM4-64 stained red spots observed with SM5-treated cells in confocal microscopy (Fig. 3E) appear to be outer membrane vesicles of approximately 100 to 300 nm diameter (Fig. 3S). Smaller outer membrane vesicles of approximately 20 to 70 nm were also observed covering the surface of the bacteria. SM1-treated cells were distorted (Fig. 3Q), while 1% DMSO-treated cells were cylindrical with no deformation (Fig. 3P), suggesting that SM1 might also weaken and disrupt the cell wall conformation of *S*. Typhimurium in addition to disrupting the cell membrane. The cell surface of SM4-treated bacteria looked roughened and crumpled (Fig. 3R). No SEM analysis was performed with SM3 due to limitation in compound availability; however, given that SM3 and SM4 have very similar chemical structures, we expect SM3 to possess a phenotype similar to that of SM4. These observations strongly suggest that the SMs alter *Salmonella* cell membrane and cell wall integrity. These conclusions were further supported by measuring the crystal violet uptake (Fig. S2A) and leakage of materials assessed at 260 nm (Fig. S2B) after 1 hr of treatment with a lethal dose of SMs. SM5-treated cells had an increase in permeability (2.32-fold) accompanied by a more abundant quantity of 260 nm-absorbing material (5.25-fold) compared to the 1% DMSO-treated cells. These results were very similar to those for cells treated with 0.25 M of ethylenediaminetetraacetic acid (EDTA), supporting the effect on *S*. Typhimurium cell membrane by SM5. However, SM1-, SM3-, and SM4-treated cells displayed an increase in 260 nm-absorbing material (2.18, 7.17, and 15.95-fold, respectively) compared to the 1% DMSO control, and showed a reduction of crystal violet uptake (1.88, 4.46, and 2.01-fold, respectively) in the treated cells compared to the 1% DMSO control. These results might be explained by the disruption of cell membranes by SM1, SM3, and SM4, as observed by confocal microscopy (Fig. 3B–D), allowing less material to be stained by crystal violet.

### SMs exhibited minimal toxicity in eukaryotic models

**Figure 4:**
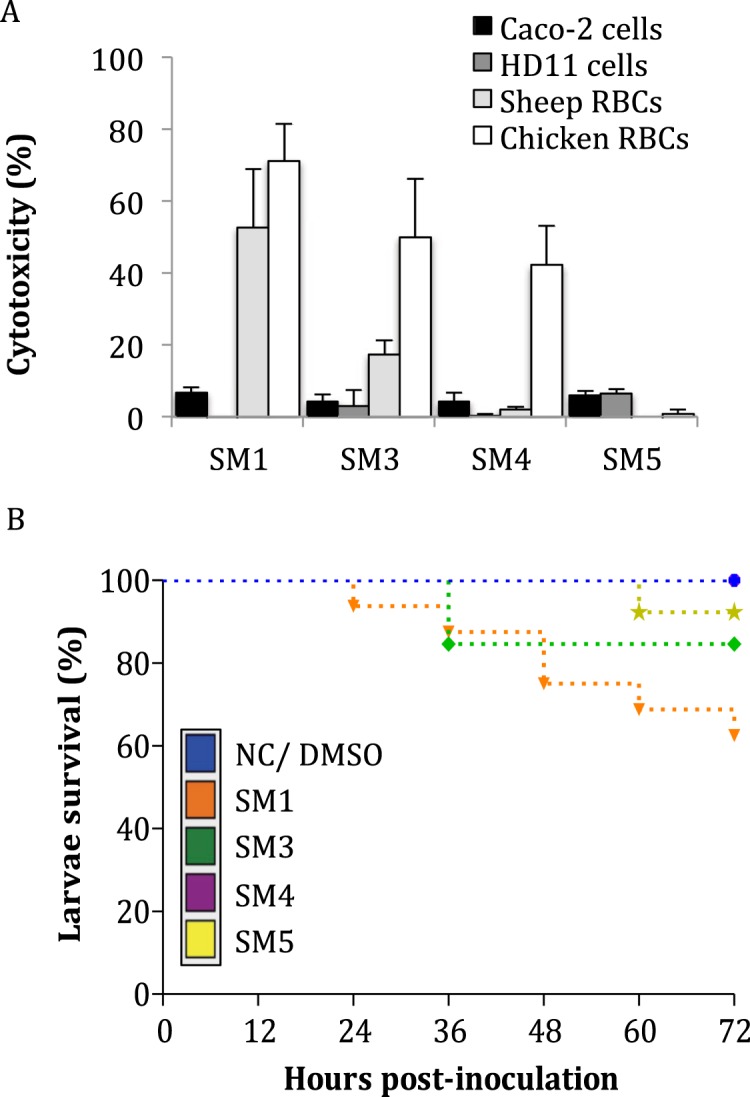
Toxicity of the four selected small molecules (SMs) on several eukaryotic models. (**A**) Cell toxicity and hemolytic activity of the SMs after 24 hrs and 1 hr treatment with 200 μM, respectively. Data were normalized with a 0.1% triton-100X control. Bar: standard deviation; n = 8 replicates per group. (**B**) *Galleria mellonella* larva survival rate after a single treatment with 12.5 μg of SM per larva. Larva death was monitored every 12 hrs for three days after treatment. Both untreated larvae (NC) and larvae treated with 1% dimethyl sulfoxide (DMSO) had 100% survival (blue line). RBCs: red blood cells; n = 15 replicates per group.

After 24 hrs of treatment with 200 µM of SMs, cytotoxicity levels were below 10% for Caco-2 epithelial cells and below 18% for HD11 macrophage cells with the all four SMs (Fig. 4A). After 1 hr treatment on sheep and chicken red blood cells (RBCs) with 200 µM of SMs, SM5 displayed a hemolytic activity lower than 1% for both RBCs; while SM3 and SM4 had a hemolytic activity below 18% for sheep RBCs and below 49% for chicken RBCs. SM1 displayed high hemolytic activity for both RBCs (>50%; Fig. 4A). Additional toxicity studies were performed in a *G. mellonella* larvae model (Fig. 4B). At 72 hrs post-infection (HPI) following a single treatment with 12.5 µg of SMs, SM4 had no lethal effect on the larva (100% survival), SM3 and SM5 displayed 85% and 92% survival, respectively, and SM1 had the most toxic effect on larvae (66% survival).

### SMs reduced intracellular *S*. Typhimurium in eukaryotic models

The ability of the four SMs to reduce *S*. Typhimurium varied in infected Caco-2, HD11, and THP-1 cell lines depending on the SMs and the cell line used (Table 3). SM3 and SM4 cleared internalized *Salmonella* at 50 µM and 25 µM, respectively in all three cell lines, while SM1 and SM5 efficacy ranged between 12.5 µM and 100 µM depending on the cell lines.

**Table 3:** Dose-response of the four selected small molecules (SMs) on *Salmonella* *enterica* subsp. *enterica* serotype Typhimurium in cell lines.

SMs	Caco-2 cells		HD11 cells		THP-1 cells	
	MBC (μM)	IC_50%_ (μM)	MBC (μM)	IC_50%_ (μM)	MBC (μM)	IC_50%_ (μM)
SM1	25	12.5 < X < 25	6.25	X < 3.215	100	X < 50
SM3	50	12.5 < X < 25	50	X < 25	50	25 < X < 50
SM4	25	12.5 < X < 25	25	12.5 < X < 25	25	12.5 < X < 25
SM5	12.5	6.25 < X < 12.5	25	X < 12.5	50	X < 25

MBC: minimal bactericidal concentration; IC_50%_: 50% inhibitory concentration; X: estimated IC_50%_.

**Figure 5:**
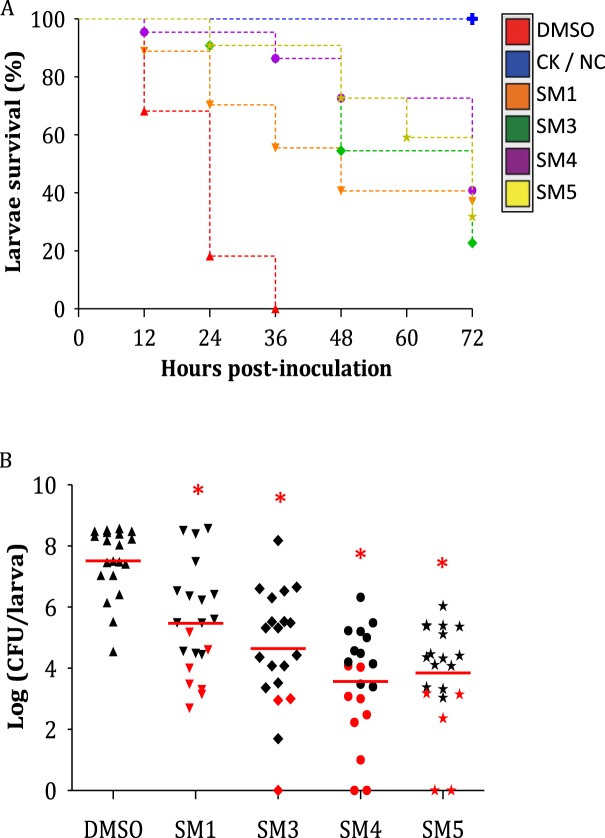
Effects of the small molecule (SM) treatments on *Salmonella* *enterica* subsp. *enterica* serotype Typhimurium infected *Galleria mellonella*. (**A**) Larva survival rate. Larva survival rate was monitored every 12 hrs for three days. Larvae not infected and not treated (NC), and infected larvae treated with 50 mg/kg chloramphenicol (CK) had 100% survival (blue line). (**B**) Bacterial quantification of *Salmonella* inside the larvae. Red dot: the larva was still alive after 72 hrs post-inoculation (HPI); black dot: the larvae that died during the experiment; n = 20; red line: mean; asterisk: internalized *Salmonella* population was significantly lower compared to the 1% dimethyl sulfoxide (DMSO) control (P < 0.01).

The *in vivo* clearance efficacy of the four SMs was also tested in *Salmonella*-infected *G. mellonella* larvae (Fig. 5A). For this experiment, a Kan^R^ *S*. Typhimurium strain was used as inoculum. Preliminary data showed that Kan^R^ *S*. Typhimurium displayed similar growth rate compared to WT *S*. Typhimurium *in vitro* (see Supplementary Fig. S3A) and the transposable element insertion was stable in *Salmonella* (Fig. S3B); Further, no differences in bacterial abundance and larva survival profile were observed with Kan^R^ *S*. Typhimurium compared to WT *S*. Typhimurium when injected to *G. mellonella* (see Supplementary Fig. S3C & D). Most of the *G. mellonella* larvae died in 24 to 36 hrs when the larvae were infected in the pro-leg with 8.5 × 10^3^ bacteria per larva, which was the minimal bacterial concentration needed to assure repeatable data and a slow larva death (see Supplementary Fig. S3E). Further the antimicrobial efficacy of the four SMs was similar between the Kan^R^ and WT *S*. Typhimurium strains in M9 medium (Table 1). To study the efficacy of the SMs in *Salmonella*-infected larvae, the SMs were injected 2 hrs before *Salmonella* infection (see Supplementary Table S3)^23^. The larval survival rate was significantly increased compared to the DMSO group when larvae were pre-treated with 12.5 µg of SMs (P < 0.01; Fig. 5A). At 24 hrs post infection (HPI) only 20% of the infected larvae pre-treated with DMSO were alive, while larvae pre-treated with the SMs showed a survival rate between 70% to 95%. All larvae from the DMSO group died by 36 HPI; however, between 25% and 45% of the larvae were still alive 72 HPI depending on the SM treatment. Moreover, SM-treated infected larvae displayed a significant reduction in *Salmonella* abundance inside the larvae (up to 4.1-log reduction) compared to the DMSO control (P < 0.01; Fig. 5B).

### SM4 and SM5 reduced *S*. Typhimurium load in chicken ceca and the colonization of systemic organs

**Figure 6:**
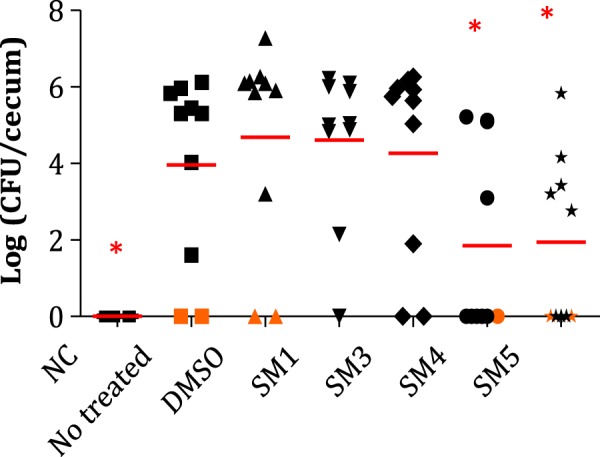
Impact of the small molecule (SM) treatments on *Salmonella enterica* subsp. *enterica* serotype Typhimuriumpopulations in chicken ceca. Red line: average; orange dots represent ceca samples that were detected positive for *Salmonella* after enrichment in tetrathionate broth; n = 10; red line: mean; asterisk: *Salmonella* populations significantly lower in ceca compared to the 1% dimethyl sulfoxide (DMSO) control (P < 0.01); NC: not treated not infected chickens; Not treated: infected chickens not treated; DMSO: infected chickens treated with DMSO; SM1, SM3-SM5: infected chickens treated with one of the four selected SMs.

After five days of treatment with 200 µg of SM4 or SM5 per day (Supplementary Table S4), one-week-old *Salmonella*-infected layer chickens displayed approximately 2.8-log reduction in *Salmonella* population inside the ceca (Fig. 6). Furthermore, chickens treated with SM4 or SM5 displayed 60% and 30% reduction of cecal colonization, and 50% and 25% reduction of spleen colonization by *Salmonella*, respectively, compared to the DMSO-treated group (Table 4). Chickens treated with SM1 and SM3 did not show any reduction of *Salmonella* in the ceca and more systemic tissues were positive for *Salmonella* in these two groups compared to the DMSO group.

**Table 4:** Tissues positive for *Salmonella* *enterica* subsp. *enterica* serotype Typhimurium after enrichment following treatment with small molecules (SMs).

Groups	Ceca (%)	Spleens (%)	Livers (%)
SM1	90	50	40
SM3	80	50	60
SM4	40	20	30
SM5	70	30	40
DMSO	100	40	40

### SM4 and SM5 had minimal impact on the cecal microbiota of chickens

**Figure 7:**
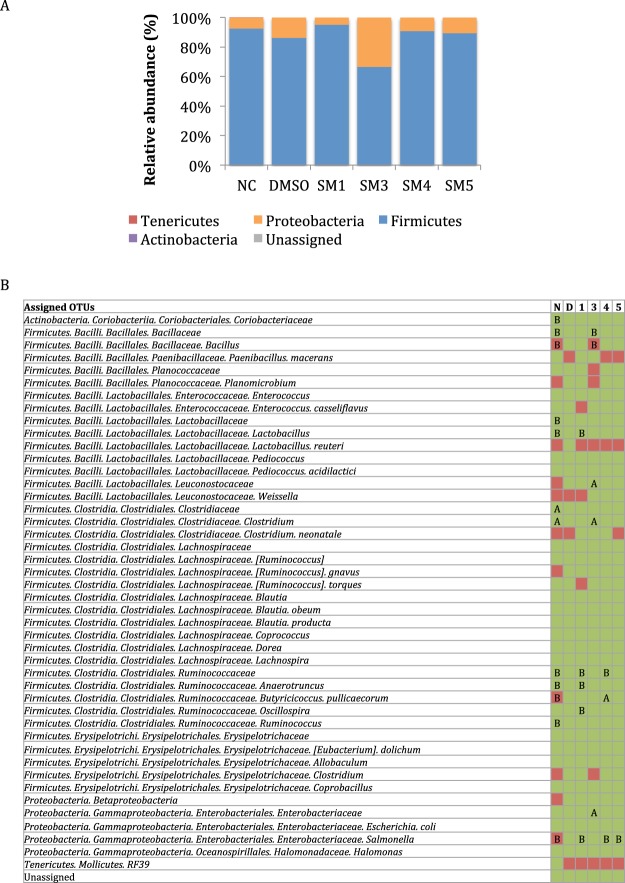
Impact of the small molecule (SM) treatments on microbiota diversity and relative abundance in chicken ceca. (**A**) Relative abundance at the phylum level. (**B**) Taxonomic diversity and significant differences between treatments in the cecal microbiota. In green: operational taxonomic units (OTUs) detected in the chicken ceca; in red: OTUs not detected in chicken ceca; A and B indicate whether the OTUs were significantly lower or higher, in abundance, respectively, compared to the 1% dimethyl sulfoxide (DMSO) group (P < 0.01); N and NC: not infected, not treated; D: DMSO-treated; 1, 3, 4, 5: treated with SM1, SM3, SM4, or SM5, respectively.

After processing of the reads and taxonomic assignment with the Greengene reference database, 1,155,383 sequences were obtained for a total of 37 samples. The number of reads per sample ranged between 24,748 and 43,688 (mean = 31,227). Analysis of the alpha diversity displayed no significant differences in the phylogenetic diversity and richness (see Supplementary Fig. S4A & B) between SM-treated groups and the DMSO control group (P > 0.01). However distinct spatial separations of the cecal samples were detected between the not treated not infected (NC) group and the five infected chicken groups when the principal coordinate analysis (PCoA) was performed with the unweighted uniFrac data (see Supplementary Fig. S4C). No distinct spatial separation was observed between the infected chickens treated with DMSO and the infected chickens treated with the SMs, suggesting that the presence of either *Salmonella* and/or DMSO altered the microbiota composition in the ceca. (see Supplementary Fig. S4C). This hypothesis was supported by the study of relative abundance at different taxonomic levels. *Firmicutes* (66% ± 8,1% to 94% ± 3,1%) and *Proteobacteria* (5% ± 3,2% to 33% ± 8,2%) represented the majority of the cecal microbiota in all chicken groups (Fig. 7A). A slight increase (6.4%) in *Proteobacteria* and a decrease (6.8%) in *Firmicutes* were observed in the DMSO group compared to the NC group. The increase in *Proteobacteria* was explained by higher abundance in *Enterobacteriaceae* (2-fold; P < 0.01; Fig. 7B); while the decrease in *Firmicutes* in the DMSO group was caused by lower abundances in *Clostridium* (25-fold), *Ruminococcus* (2-fold), *Coprococcus* (2-fold), and a small reduction of the other OTU abundances within *Firmicutes* compared to the NC group (P < 0.01). However, the DMSO group was also characterized by significant increases in *Lactobacillus* (663-fold), *Anaerotruncus* (2.3-fold), *Ruminococcus* (3.5-fold), and in *Coriobacteriaceae* (32-fold) compared to the NC group (P < 0.01; Fig. 7B).

Different microbiota profiles were also observed between the DMSO- and SMs*-*treated, *Salmonella* infected groups (Fig. 7A). A reduction in *Salmonella* OTUs was detected in chickens treated with SM1, SM4, or SM5 compared to the DMSO group (P < 0.01; Fig. 7B), which was also observed in the bacterial counts (Fig. 6) for chickens treated with SM4 or SM5, but not SM1. Given the *Salmonella* OTU population represented approximately 0.003% of the total microbiota, the difference in sensitivity and specificity of the two techniques used might be the cause of divergent results for SM1. SM4 treatment also decreased the *Ruminococcaceae* abundance while increasing *Butyricicoccus pullicaecorum* compared to the DMSO control (P < 0.01; Fig. 7B). On the other hand, the SM1-treated group had a lower abundance of *Proteobacteria* and a higher abundance of *Firmicutes* compared to the DMSO group due to a general increase and decrease of most OTUs in *Firmicutes* and *Proteobacteria*, respectively (P < 0.01). Microbiota of SM1-treated chickens was also characterized by significant decreases in *Ruminococcaceae* and *Lactobacillus* OTUs. Infected chickens treated with SM3 displayed a lower abundance of *Firmicutes* compared to the DMSO control, which was explained by a general decrease of the *Firmicutes* OTUs as well as a significant decrease of *Bacillales* OTUs, and an increase in *Proteobacteria*, resulting from a significantly higher level of *Enterobacteriaceae* (2.5-fold) compared to the DMSO control (P < 0.01; Fig. 7B). Further, the SM3-treated group was also characterized by a significant increase in OTUs of *Leuconostocaceae* (10-fold) and *Clostridium* (15-fold; P < 0.01).

## Discussion

Despite the progress in controlling *Salmonella* from farm to fork in the recent years, *Salmonella* is still a persistent problem on poultry farms. In the current study, we identified two novel SM (SM4 and SM5) growth inhibitors effective against *Salmonella* in chickens, which may facilitate the mitigation of *Salmonella* from farm to fork. The identification of *Salmonella* growth inhibitors was initiated through the screening of 4,182 bioactive SMs against *S*. Typhimurium LT2 strain at 200 µM. Initial screening using rich media yielded only very few hits (n = 8); the number of growth inhibitors identified as well as their antimicrobial efficacy towards *Salmonella* were increased when the bacteria were challenged in minimal growth conditions, suggesting that nutrient availability is a crucial parameter for *Salmonella* to resist antimicrobials^24^. Nutrients regulate important bacterial physiological processes such as cells division, cell size, and numerous metabolic pathways, which lead to weaker defense mechanisms when bacteria are in an environment with limited nutrient resources^24,25^.

Of the 19 SMs that completely inhibited *S*. Typhimurium growth in minimal growth conditions, four SMs were effective at a low concentration (10 µM and higher) against several serotypes, biofilm embedded, and antibiotic-resistant *Salmonella*, as well as other bacterial pathogens such as avian pathogenic *E. coli*, EHEC O157:H7, *C. jejuni*, *L. monocytogenes*, and *M. gallisepticum*. These four novel antimicrobial agents could represent valuable treatments against emerging AMR *Salmonella* and could also be used to control other poultry and foodborne pathogens on poultry farms, and in products. Furthermore, these four SMs had synergetic and additive effects on the antimicrobial activity of antibiotics (ciprofloxacin, meropenem, and cefeprime) currently used to control *Salmonella* in animal production and human infections. For example, the combination of a sub-lethal dose of both SM1 and ciprofloxacin reduced the ciprofloxacin MBC from 0.0625 µg/ml to 0.001 µg/ml. A reduction in antibiotics’ uses would contribute to mitigation and also reduce the emergence of antibiotic-resistant bacteria, thereby reducing the economic burden associated with infections due to antibiotic-resistant bacteria^26,27^. It was also found that at 200 µM, SM5 did not affect the growth of *Bifidobacterium longum*, *Lactobacillus brevis*, and *Lactobacillus rhamnosus GG*, which are known to have anti-*Salmonella* effects (data not shown)^28–30^. Thus, SM5 could be combined with these probiotics to enhance the control of *Salmonella* in poultry production systems. The combination of two control methods would also reduce the risk for *Salmonella* developing resistance to these complementary approaches.

All four SMs disrupted the *Salmonella* cell membrane when the bacteria were challenged with a lethal dose of SMs, causing significant leakage of cellular content^31^. Specifically, SM1, SM3, and SM4 affected *S*. Typhimurium cell membrane integrity. A previous study showed a similar effect when *E. coli* was treated with peptoids containing tryptophan-like side chains, which have strong affinity for membranes^32^. SM1 and SM5 contain a benzimidazole group, an analogue of tryptophan; and SM3 is composed of an imidazole group, which is a component of a benzimidazole group^33^. Similar phenotypic alteration of the membrane integrity was also described when *E. coli* was treated with PMAP-36, melitin, gramicidin, peptidyl-glycylleucine-carboxyamide, nisin, carvacrol, and cinnamon, which were associated with the disruption and depolarization of the cell membrane, and detrimental effects on the cell wall integrity^34–36^. These results suggest that the three SMs might interact with the *Salmonella* cell membrane and affect its integrity. This hypothesis could also explain the broad spectrum of activity of these compounds as the cell membrane composition is conserved between bacteria^37^. Further, a recent study focusing on molecules having high structural similarities with SM3 and SM4 (ionic liquids composed of a hexadecyl group and imidazole derivatives) showed that these molecules reduced the growth, adhesion, and biofilm formation in *Navicula* sp. algae^38^. Further, based on cytological studies they also hypothesized that these inhibitions were caused by a disruption of the cell membrane^38^. Several ionic liquids are known to have anti-biofilm activities^39^. Both SM3 and SM4 were effective against biofilm embedded *S*. Typhimurium.

SM5 treatment resulted in unusual bleb/outer membrane vesicles in every bacterium. A similar phenotype has been previously reported in *E. coli* treated with JB-95, a β-hairpin macrocyclic peptide^40^. JB-95 has been shown to disrupt the outer membrane of *E. coli*, causing an accumulation of membrane-like structure in the periplasm and formation of knoblike protuberances outside the cell. The same phenotype was also observed when the 191–586 region of the *pbgA* gene (also named *yejM*) was deleted in *Salmonella*^41^. PbgA is an inner membrane protein allowing, in a PhoPQ-dependent manner, the movement of cardiolipins in the outer membrane, which is essential for bacterial pathogenesis and survival^41–43^. In addition, previous high-throughput screening (PubChem bioassay AID #1863, #1981, #2253, and #2401; https://pubchem.ncbi.nlm.nih.gov/compound/9586485#section=BioAssay-Results) studies have identified that SM5 is a direct or indirect inhibitor of the PhoP regulon in *S*. Typhimurium. Taken together, these results might suggest that SM5 kills *Salmonella* by potentially affecting the cardiolipin organization in the outer membrane. This hypothesis is further supported by a significant increase of *S*. Typhimurium cell membrane permeability (crystal violet uptake and release of 260 nm absorbing material into supernatant; see Supplementary Fig. S2) after challenge with a lethal dose of SM5.

Of the four SMs tested against *Salmonella* in infected chickens, SM4 and SM5 successfully reduced the *Salmonella* population in ceca (approximately 2.8-log reduction), as well as the systemic spread of the pathogen to the liver, with minimal impact on the cecal microbiota. On the other hand, SM1 and SM3 displayed antimicrobial effects against *Salmonella* in cell culture and in *G. mellonella* but not in chickens. The lack of anti-*Salmonella* activity in infected chickens treated with SM1 and SM3 might be caused by several factors including disturbance of the microbial population in the chicken ceca as SM1 resulted in a reduction of the *Proteobacteria*/*Firmicutes* ratio while SM3 increased the ratio compared to the DMSO control (Fig. 7A). The microbiome acts as a bridge communicating what happens inside the gut and also as a moderator of metabolism^44,45^. An alteration of gut bacteria diversity influences the degradation of complex molecules and the production of metabolites, and in consequence modulates the resistance of the host toward enteric pathogen colonization^46–48^.

Most of the microbiota variations detected in the SM4- and SM5-treated chickens’ ceca were within the *Clostridiales* and *Lactobacillales* orders. *Clostridiales* bacteria have been identified as major actors in short chain fatty acid (SCFA) metabolism, resulting in the production of butyrate, propionate, or acetic acid^49,50^. These metabolites act as growth performers or host defense stimulators, and are effective control methods against enteric pathogens in chickens^49–52^. For example, the use of butyrate or butyrate producers such as *Butyricicoccus pullicaecorum*, which was significantly enhanced in SM4-treated chickens, has been described as effective in controlling *Salmonella*, *Campylobacter*, and *Clostridium perfringens* in layer chickens^53–55^. Based on these results, we propose that the reduction of *Salmonella* observed in infected chickens treated with SM4 is related to the anti-*Salmonella* activity of the SMs and its growth-promoting effects on *Salmonella*-antagonistic microbes such as *Butyricicoccus*. Butyrate is also an important regulator of tight junction proteins (TJP), which are involved in the permeability between lumen and hepatic cells. Therefore reduction in the systemic colonization of the host observed with SM4-treated chickens could be explained by an improvement in intestinal barrier functions^56^. This hypothesis might also corroborate results obtained with SM5-treated chickens. Despite the observation that both SM5 and SM4 treatments displayed similar *Salmonella* survival rates in ceca, SM4 had a more significant reduction of systemic translocation of the bacteria than SM5-treated chickens. Further, the use of DMSO to counter the solubility issues with the SMs during the chicken experiment also altered the ceca microbiota, which could explain the higher abundance of *Salmonella* in the presence of DMSO. Our future studies will focus on using other solvents with minimal impact on the microbiota^57^.

In summary, two novel SMs (SM4 and SM5) effective in controlling *Salmonella* in poultry were identified in this study. These SMs had no or minimal impact on chicken cecal microbiota. These SMs can be also utilized against other poultry and foodborne pathogens, and showed compatible utilization with other anti-*Salmonella* strategies such as antibiotics (ciprofloxacin, meropenem, and cefeprime) and probiotics (*Lactobacillus*). However, the SMs display some limitations concerning the mass application in poultry production due to their insolubility at high concentrations in water. Therefore, future studies focusing on the 1) creation of derivatives to improve water solubility, 2) enhance bioavailability by administering with suitable solubilizers or carriers, and 3) identify their specific drug target in *S*. Typhimurium. These studies will facilitate development of these compounds for commercial applications.

## Materials and Methods

### Bacterial strains and growth conditions

*Salmonella enterica* subsp. *enterica* serovar Typhimurium LT2 strain (*S*. Typhimurium wild-type; WT) was used as the primary strain for the identification of growth inhibitors. Additional *Salmonella* serovars, antimicrobial-resistant *Salmonella* strains, and foodborne and avian pathogens were used for activity spectrum characterization. A kanamycin-resistant *S*. Typhimurium strain (Kan^R^ *S*. Typhimurium) was used for the efficacy studies with *Galleria mellonella* larvae and chickens. Details of the bacterial strains used and their growth conditions are listed in Supplementary Table S2.

### Eukaryotic models used in this study

Three cell lines (Caco-2, HD11, and THP-1) and *G. mellonella* (wax moth) larvae were used to evaluate the cytotoxicity and *Salmonella* clearance ability of the SMs. One-week-old layer chickens were used as proof of concept to validate the anti-*Salmonella* efficacy of the four SMs in poultry and their impact on the cecal microbiota in chickens. Details of the organisms used and their growth conditions are listed in Supplementary Table S2.

### SM library

A library of 4,182 bioactive SMs obtained from ChemBridge (San Diego, CA, USA) was used^58^. The SMs were suspended into 100% dimethyl sulfoxide (DMSO) to 10 mM concentration and stored at −80 °C in 96-well plates sealed with Thermowell seal tape (Corning).

### Creation of Kan^R^ S. Typhimurium mutant

The kanamycin-resistant (Kan^R^) *S*. Typhimurium strain was created using the pUWGR4 plasmid carrying EZ::TN transposable element as previously described^59^. The WT *S*. Typhimurium electro-competent cells were prepared as described in the Bio-Rad manual (#3112_54). An ice-cold electroporation cuvette (Eurogentech; 2 mm gap) was transferred with 2 µl of the transposable kanamycin gene construct plus 100 µl of competent cells. Cells were transformed with a MicroPulser (Biorad) at 2,400 V, 25 µF, and 400 Ω. Immediately after electroporation, 900 µl SOC medium was added, and the mixture was transferred into a 1.5 ml Eppendorf^®^ tube incubated at 30 °C, 180 rpm for 90 min. The suspension was then plated on XLT-4 agar plates supplemented with 50 µg/ml kanamycin and incubated up to three days at 37 °C. A colony polymerase chain reaction assay was performed on one of the colonies obtained to confirm the insertion of the Kan^R^ gene (837 base pairs) as previously described^59^. Then the *in vitro* insertion stability of the EZ::TN transposon was tested as previously described^60^ by serial passaging overnight (12 hrs) at 37 °C without antibiotic for 10 times (approximately 50 generations).

### Identification of S. Typhimurium growth inhibitor SMs

The 4,182 SMs were screened at 200 µM against *S*. Typhimurium using high-throughput screening assay in a 96-well plate format^61^. An overnight *Salmonella* suspension was normalized to 0.05 OD_600_ (approximately 3.5 × 10^7^ CFU/ml) with M9 minimal broth medium supplemented with 0.05% casamino acids and 0.4% glucose^62^. One hundred microliter of the suspension plus 2 µl of SMs (200 µM) were transferred into each well of a sterile, non-treated, flat bottom 96-well plate. Bacteria alone (negative control, NC), 2% DMSO plus bacteria (DMSO control), 20 µg/ml chloramphenicol or 50 µg/ml kanamycin plus bacteria (positive controls, PC), and M9 medium only were used as controls. Plates were incubated in a Sunrise Tecan kinetic microplate reader for 12 hrs at 37 °C and the optical density (OD) was measured at 600 nm. The percentage of growth inhibition was calculated as: [(OD_600 SM_ − OD_600 NC_)/OD_600 NC_] × 100. Cultures from wells showing no turbidimetric increase were transferred onto a XLT4 agar plate. Plates were incubated at 37 °C for 36 hrs.

### SMs activity spectrum

The 128 SMs inhibiting *S*. Typhimurium growth (20% or higher) were tested for their antimicrobial effect on nine different *Salmonella* serovars frequently implicated in foodborne gastroenteritis outbreaks. Further, the four most potent SMs (SM1, SM3 - SM5) that showed complete growth inhibition on all *Salmonella* serovars were tested for their effect on other foodborne (n = 2) and avain pathogens (n = 12). Both screens were performed at 200 µM as described in the primary screening. Growth conditions for each strain are described in Supplementary Table S2.

### MIC and MBC determination

The 19 SMs that completely inhibited *S*. Typhimurium growth were 2-fold serially diluted to obtain a final SM concentration ranging from 400 µM to 2.5 µM. *S*. Typhimurium was challenged with a determined concentration of SM as described in the primary screen. The lowest SM concentration that completely inhibited the growth without killing the bacteria was considered as MIC and the lowest SM concentration with a cidal effect was considered as MBC. A similar dose-response assay was performed with the four most potent SMs on the other *Salmonella* serovars.

### Antimicrobial resistance studies

Single step and sequential passage resistance assays were performed with the four most potent SMs (SM1, SM3-SM5) as previously described^61^. The MBC values displayed in Table 1 for S. Typhimurium were used as reference for the lethal (2X MBC) and sub-lethal (0.75X MBC) doses.

For the single step resistance assay, 10^9^ *S*. Typhimurium bacteria were plated in a well of a 24-well plate containing M9 agar supplemented with 2X MBC of SMs and incubated for 15 days in the dark at 37 °C. Then 100 µl of LB broth was added to each well to resuspend any surviving bacteria, transferred into a tube containing 5 ml of LB medium, and incubated for 12 hrs at 37 °C, shaking at 150 rpm. Tubes showing an increase in OD_600_ and any colonies that grew in the 24-well plate were tested for MIC and MBC.

For the sequential passage resistance assay: *S*. Typhimurium was challenged in a 96-well plate containing M9 medium supplemented with 0.75X MBC of SMs (concentration allowing at least 70% growth inhibition) as described in the primary screening. The 96-well plate was incubated in the dark at 37 °C, 175 rpm for 12 hrs. After the first passage, the plate was centrifuged for seven min at 4700X g, supernatant was replaced with a fresh M9 broth medium amended with 0.75X MBC of the corresponding SM and grown for 12 hrs. This procedure was repeated fourteen times. Following the 15^th^ passage, MIC and MBC were determined as described previously. For both experiments, *S*. Typhimurium grown in 2% DMSO, 20 µg/ml chloramphenicol, or 50 µg/ml kanamycin, and M9 medium only were used as controls.

### Confocal microscopy and Scanning Electron Microscopy (SEM)

An overnight suspension of *S*. Typhimurium grown in M9 medium (approximately 1 OD_600_) was washed in 1X phosphate-buffered saline (PBS), resuspended in fresh M9 medium containing a lethal dose of SMs (5X MBC) and grown for 3 hrs. The effect of the SMs on *S*. Typhimurium was assessed using confocal microscopy as previously described^31^. Cells were stained for 45 min using FM4–64 (2 µg/ml; Molecular Probes) and SYTO-9 (5 µM; Invitrogen). Three microliters of stained bacteria were transferred onto agarose (1.2%) -coated glass slides. Microscopy was performed using a Leica TCS SP6 confocal scanning microscope with FM4–64 (515 nm/640 nm) and SYTO-9 (485 nm/498 nm) filters.

Processing of the samples for SEM was performed with the same samples as above and as previously described^63^. Briefly, one volume of bacterial suspension was mixed with one volume of fixative (3% glutaraldehyde, 1% paraformaldehyde in 0.1 M potassium phosphate buffer pH 7.2), and incubated for 2 hrs at 4 °C. Fixed cells were centrifuged for 5 min at 1,200 g, washed twice with 1X PBS, and resuspended into 1% osmium tetroxide for 1 hr at room temperature in the dark, followed by serial dehydration of the sample in ethanol and platinum splatter-coating. Visualization and imaging of the samples was performed using a Hitachi S-4700 scanning electron microscope.

### Antimicrobial susceptibility testing on biofilm embedded *S*. Typhimurium using the MBEC-HTP assay

The antimicrobial efficacy of the four SMs was tested on biofilm embedded *S*. Typhimurium as previously described^64^. Briefly, 150 µl of an overnight suspension of *S*. Typhimurium normalized to 0.05 OD_600_ in LB medium was transferred into each well of a sterile, non-treated, flat bottom 96-well plate. The plate was covered using the lid containing the pegs (Innovotech), sealed with parafilm, and incubated for 36 hrs at 37 °C. After incubation, the biofilm-coated pegs were soaked in 175 µl of sterile water for 30 sec in a 96-well plate and transferred into a new 96-well plate containing 200 µl of M9 medium supplemented with 0.2X MBC to 4X MBC of SMs. The plate was incubated for 18 hrs at 37 °C, 110 rpm in the dark. After incubation, the pegs were transferred to a new 96-well plate containing 200 µl of sterile water and sonicated for 60 min at room temperature (Aquasonic model 50HT, VWR). Removal of the biofilm was confirmed by crystal violet staining. The supernatant was ten-fold serial diluted, plated on an agar plate, and incubated at 37 °C for 36 hrs. The lowest SM concentration giving a complete clearance was considered as MBEC. *S*. Typhimurium challenged with 1% DMSO or cefeprime (between 0.4X MBC to 4X MBC; MBC = 2 µg/ml) were used as controls. In parallel to this experiment, a dose-response assay with planktonic cells was conducted as described above.

### Potentiation of effect of SMs on antibiotics

The potentiation effect of several antibiotics, commonly used to control *Salmonella*, was studied in a checkerboard assay^65^. Six antibiotics (cefotaxime, cefeprime, ciprofloxacin, erythromycin, nalidixic acid, and meropenem) were tested on *Salmonella* in M9 medium. The MBC of each antibiotic was determined in M9 medium prior the experiment using a dose-response assay, as described above. For potentiation studies, 100 μl of a *S*. Typhimurium suspension normalized to 0.05 OD_600_ was transferred to each well of a 96-well plate. Different concentrations of SMs and antibiotics ranging between 0.2X MBC and 1X MBC were added to each well. The MBC of each antibiotic was determined as described in the dose-response assay. Bacteria challenged with SMs or antibiotics alone at the same concentrations were used to determine the antibiotic-SM combination effects. Bacteria alone, supplemented with 2% DMSO, or 50 µg/ml kanamycin, and M9 medium only were used as controls. Plates were incubated in a Sunrise Tecan kinetic microplate reader for 12 hrs at 37 °C and the OD was measured at 600 nm. Further, the bacterial suspension was plated on an agar medium and incubated at 37 °C for 36 hrs. The antibiotic-SM combination effect was calculated based on the determination of the fractional bactericidal concentration (FBC) as previously described^66,67^.

### Cytotoxicity of the four selected SMs on cell lines

Cytotoxicity of the four SMs was tested on Caco-2 and HD11 cells at 200 µM as previously described^68^. Briefly, a 96-well plate was seeded with 150 µl Caco-2/HD11 cells (approximately 1.4 × 10^5^ cells per well) in cell culture medium and incubated at 37 °C in a humidified 5% CO_2_ incubator. Once a confluent monolayer was formed, cells were washed three times with 1X PBS and 150 µl of growth medium supplemented with 1 µl of SMs (200 µM) was added. After 24 hrs of incubation, cytotoxicity levels were determined using the Pierce Lysine Dehydrogenase Cytotoxicity Assay Kit (ThermoFisher Scientific). One percent DMSO and 10X lysis buffer were used as controls. The cytotoxicity level was calculated according to manufacturer instructions.

### Hemolytic activity of the four selected SMs on RBCs

The hemolytic activity of the four SMs was demonstrated as previously described^69^. Briefly, 200 µl of 10% sheep or chicken RBC suspension was incubated with 1 µl of SMs (200 µM) for 1 hr at 37 °C in a 96-well plate. After incubation, the plate was centrifuged at 3700 X g for 5 min at 4 °C and then placed on ice for five min. One hundred microliters of the supernatant were transferred into a fresh 96-well plate, and the OD was measured at 540 nm. One percent DMSO and 0.1% Triton-100X were used as negative and positive controls, respectively. Percentage hemolysis was calculated as: [(OD_540 SM_ − OD_540 DMSO_)/(OD_540 1X triton_ − OD_540 PBS_)] × 100.

### Toxicity of the four selected SMs on *G*. *mellonella* larvae

*Galleria mellonella* larvae (fifth instar stage) were incubated for 12 hrs at 37 °C in the dark. After incubation, only larvae with a white creamy phenotype and a body weight ranging between 225 to 275 mg were selected for the study. Larvae (n = 15 per group) were inoculated in one of the last pro-legs with 12.5 µg of SMs (8.5 µl; 50 mg/kg) using a PB600-1 repeating dispenser (Hamilton, Reno, NV) attached to 300 µl insulin syringe, 31 gauge 8 mm needle length (ReliOn^®^, Bentonville, AR). SMs were diluted in a buffer mix (30% DMSO plus 10 mM MgSO_4_)^23^. Larvae were placed inside a plastic petri dish and incubated for 3 days in the dark at 37 °C. Larval survival was monitored on 12 hrs intervals. Not treated larvae, larvae treated with the buffer mix, and larvae treated with 12.5 µg of chloramphenicol were used as controls.

### Effect of the four SMs on *S*. Typhimurium survival in cells lines

The ability of four SMs to clear *S*. Typhimurium was evaluated using three cell lines (Caco-2 cells, HD11, and THP-1 cells) as previously described^69^. A multiplicity of infection (MOI) of 10 was used. Infected cells were treated with 1 µl of SMs (final concentration ranged between 100 µM and 6.25 µM) and incubated at 37 °C for 24 hrs in humidified, 5% CO_2_ incubator. Following incubation, cells were washed once with 1X PBS, lysed with 0.1% Triton-100X, serial ten-fold diluted in 1X PBS, and plated on agar plate. Plates were incubated at 37 °C for 24 hrs to determine the intracellular bacteria. Cells not infected and not treated, and cells infected and treated with 2% DMSO were used as controls.

### Effect of the selected four SMs on *S*. Typhimurium in *G. mellonella* larvae

Wax moths were selected as mentioned above. For this study, Kan^R^ *S*. Typhimurium was used for challenge. First, the virulence of Kan^R^ S. Typhimurium was assessed in comparison to WT parent *S*. Typhimurium strain in *G. mellonella*. Briefly, larvae (n = 20) were infected in one pro-leg with approximately 8.5 × 10^3^ *S*. Typhimurium suspended in 10 mM MgSO_4_ (8,5 µl of inoculum) and incubated for three days in the dark at 37 °C in a petri dish. Survival was monitored every 12 hrs for three days. Bacterial quantification was performed once the larvae had died (dark pigmentation or no reaction to a mechanical stimulus) or after 72 hrs of incubation. Larvae were washed once with 70% ethanol, twice with sterile distilled water for 30 sec each, and transferred individually into an Eppendorf^®^ tubes containing 1 ml of 1X PBS and homogenized. The mixture was serially diluted, plated on XLT-4 agar plate supplemented with 50 µg/ml kanamycin, and incubated for 36 hrs at 37 °C. Larvae not treated and larvae treated with the buffer mix were included as controls. The virulence of Kan^R^ and WT *S*. Typhimurium strains was assessed by comparing the larval mortality and bacterial counts.

To test the effect of SMs on *S*. Typhimurium in larvae, SMs and Kan^R^ *S*. Typhimurium were injected into two different pro-legs and at different time points as previously described^23^. First, the larvae were treated with SMs, then incubated for 2 hrs at 37 °C in a petri dish, and infected with approximately 8.5 × 10^3^ of Kan^R^ *S*. Typhimurium per larva. Summary of treatments are described in Supplementary Table S3. Larvae treated with 50 mg/kg chloramphenicol were used as control (lowest concentration of antibiotic allowing 100% larva survival rate; see Supplementary Fig. S3F).

### Effect of selected SMs on the survival of *S*. Typhimurium in one-week-old layer chickens

One-week-old *Salmonella*-free layer chickens were orally inoculated with approximately 10^4^ Kan^R^ *S*. Typhimurium. Rectal swabs were collected to confirm the intestinal colonization by *Salmonella* prior to treatment. At 3 days post infection (DPI), chickens were treated orally twice a day for five days with SMs (100 µg per chicken). Details of the treatment groups are described in Supplementary Table S4. Following treatment, chickens were euthanized and tissues were aseptically collected (ceca, liver, and spleen). One cecum per pairs was immediately stored at −80 °C for microbiota studies. Ceca, spleens, and liver tissues were suspended in 1X PBS and homogenized. One milliliter of the undiluted homogenized tissue was enriched in 9 ml of tetrathionate broth for 18 hrs at 37 °C. The remaining homogenized tissues were serially ten-fold diluted, plated on XLT4 agar plate supplemented with 50 µg/ml kanamycin, and incubated for 36 hrs at 37 °C. The chicken experiment was approved by The Ohio State University Animal Care and Use Program (accredited by the Association for Assessment and Accreditation of Laboratory Animal Care International) and performed following the Institutional Animal Care and Use Committee (IUACUC) protocol n° 2010A00000149-R2-AM1.

### DNA extraction and 16S sequencing

Genomic DNA was extracted from ceca using the *PureLink Microbiome DNA* Purification *Kit* (Life Technologies, Invitrogen Corp.), combined with RNAse treatment (10 units/hr). About 0.15 g to 0.20 g of cecal content was used for DNA extraction. After quality control with electrophoresis and nanodrop, extracted DNA samples were subjected to 16S rRNA V4-V5 variable region sequencing. Amplicon libraries were prepared by using Phusion® High-Fidelity PCR Kit (New England Biolabs Inc, Ipswich, MA) as previously described^70^. PCR products were cleaned using AMPure XP PCR (Beckman Coulter Inc, Beverly MA) and sequenced using Illumina MiSeq 300-base, paired-end kit at the Molecular and Cellular Imaging Center (https://mcic.osu.edu/).

### Bioinformatics analyses

Quality control of the raw reads was performed using FastQC (Babraham Bioinformatics, Cambridge, USA). Only nucleotides with a base sequence quality whose median quality score was above 25 and whose lower quartile median quality score was above 10 were used for further analysis. Trimmomatic was used for trimming and removal of NexteraPE-PE adapter sequences^71^ (http://mcbl.readthedocs.io/en/latest/mcbl-tutorials-AD-clean.html). The resulting forward and reverse sequences were merged using Pandaseq (https://github.com/neufeld/pandaseq). Any sequence with less than 0.7 threshold overlap was removed and spacers used for amplification were trimmed. Samples were processed using Quantitative Insights Into Microbial Ecology (QIIME) software version 1.9^72^. Operational Taxonomy Units (OTUs) were determined by clustering reads against Greengenes 16S reference dataset (2013–08 release) at a 97% identity using an open-reference OTU picking (pick_open_reference_otus.py) method using default parameters, except setting minimum OTU size to 10. Microbial diversity was studied after rarefication of the sequences based on the lowest number of sequences among the samples tested (n = 14,000). Alpha and beta diversities were analyzed using the core analysis package (core_diveristy_analyses.py), which included the comparison of the phylogenetic diversity and richness, PCoA, and relative abundance studies. A weighted UniFrac distance matrix was generated from the open OTU picking results and was visualized in a PCoA plot using the EMPeror program. The identification of microbial relative abundance differences between treatments was performed using linear discriminant analysis (LDA) in the Galaxy|Hutlab website (https://huttenhower.sph.harvard.edu/galaxy/).

#### Chemical structure analysis of the SMs

The physico-chemical properties of SMs were analyzed using PubChem Compounds (National Center for Biotechnology Information; Rockville Pike, MD) and ChemMine website (Backman *et al*., 2011; Bolton *et al*., 2008). SMs were clustered based on their structural similarities. A Tanimoto score was calculated from a two-dimensional (2D) structure fingerprint using a single linkage algorithm.

#### Statistical analysis

Growth curves, bacterial counts, crystal violet uptake and 260 nm absorbing material data were analyzed using JMP PRO 12 software (SAS Institute, Cary, NC, USA). A one-way ANOVA combined with a Student T-test was used to assess the difference between the treatments. Statistical analyses of the *G. mellonella* survival data were performed in GraphPad Prism 5 software (GraphPad, Inc., CA, USA) using the Kaplan-Meier estimator^73^. Analysis of the OTU relative abundance between treatments was analyzed in the Galaxy|Hutlab website using a linear discriminant analysis effective size (LefSe). A Kruskall-Wallis test combined with a pairwise Wilcoxon test was performed to identify statistical differences^74^. Correlations in relative abundance between specific OTUs within each treatment were studied using a bivariate analysis in JMP Pro 12 software. For each statistical analysis, a p-value ≤ 0.01 was considered as statistically significant^75^.

### Ethic statement

The chicken experiment was approved by The Ohio State University Animal Care and Use Program (accredited by the Association for Assessment and Accreditation of Laboratory Animal Care International) and performed following the Institutional Animal Care and Use Committee (IUACUC) protocol n° 2010A00000149-R2-AM1.

## Electronic supplementary material

Supplmental Table S1-S4

Supplmental Figures S1-S4
