# Author Correction: Novel Imidazole and Methoxybenzylamine Growth Inhibitors Affecting *Salmonella* Cell Envelope Integrity and its Persistence in Chickens

**DOI:** 10.1038/s41598-019-52826-x

**Published:** 2019-11-06

**Authors:** Loïc Deblais, Yosra A. Helmy, Dipak Kathayat, Huang-chi Huang, Sally A. Miller, Gireesh Rajashekara

**Affiliations:** 1 Food Animal Health Research Program, Department of Veterinary Preventive Medicine, The Ohio State University, OARDC, Wooster, OH USA; 2 Department of Plant Pathology, The Ohio State University, OARDC, Wooster, OH USA

Correction to: *Scientific Reports* 10.1038/s41598-018-31249-0, published online 06 September 2018

The original version of this Article contained a typographical error in the spelling of the author Dipak Kathayat, which was incorrectly given as Diapak Kathayat. This error has now been corrected in the PDF and HTML versions of the paper, and in the accompanying supplementary material.

