# Supplementary material for: Novel Imidazole and Methoxybenzylamine Growth Inhibitors Affecting Salmonella Cell Envelope Integrity and its Persistence in Chickens
Source: Sci Rep. 2018 Sep 6;8:13381. doi: 10.1038/s41598-018-31249-0 (PMC6127322; doi:10.1038/s41598-018-31249-0)
Supplement: Supplementary file 1 — Supplmental Table S1-S4 [file 41598_2018_31249_MOESM1_ESM.pdf]

Novel Imidazole and Methoxybenzylamine Growth Inhibitors Affecting *Salmonella* Cell Envelope Integrity and its Persistence in Chickens

Loïc Deblais[1,2], Yosra A. Helmy[1], Dipak Kathayat[1], Huang-chi Huang[1], Sally A. Miller[2], Gireesh Rajashekara[1*].

*Address correspondence to Dr. Gireesh Rajashekara, rajashekara.2@osu.edu.

[1]Food Animal Health Research Program, Department of Veterinary Preventive Medicine, The Ohio State University, OARDC, Wooster, OH.

[2]Department of Plant Pathology, The Ohio State University, OARDC, Wooster, OH.

Supplemental Table 1. Chemical information about the selected 19 SMs that completely inhibited *S*. Typhimurium growth

SM	Pubchem ID	Chemical name	Molecular weight	cLogP	Follow the rule of five
SM1	2834410	1,3-bis{2-[(2-isopropyl-5-methylcyclohexyl)oxy]-2-oxoethyl}-1H-3,1-benzimidazol-3-ium chloride	547.2	5.56	No
SM2	2836168	1-(3,6-dibromo-9H-carbazol-9-yl)-3-[(2-hydroxyethyl)(methyl)amino]-2-propanol	456.2	4.66	Yes
SM3	2847561	1-(cyanomethyl)-3-hexadecyl-1H-imidazol-3-ium chloride	368	8.75	No
SM4	2848076	1-hexadecyl-3-methyl-1H-3,1-benzimidazol-3-ium bromide	437.5	10.19	No
SM5	9586485	N''-{4-[(4-chlorobenzyl)oxy]-3-ethoxybenzylidene}carbonohydrazonic diamide	346.8	4.58	Yes
SM6	16192520	1-[benzyl(methyl)amino]-3-[4-({[(5-chloro-2-thienyl)methyl]amino}methyl)phenoxy]-2-propanol	431	4.118	Yes
SM7	5333224	2,4-dichloro-6-{[(2-fluoro-5-nitrophenyl)imino]methyl}phenol	329.1	3.96	Yes
SM8	5333245	2,4-dichloro-6-{[(3-methoxyphenyl)imino]methyl}phenol	296.1	3.68	Yes
SM9	45215046	N-[1-(4-isobutylbenzyl)-3-piperidinyl]-2-(4-methyl-1,4-diazepan-1-yl)acetamide	400.6	3.948	Yes
SM10	597279	ethyl 5-acetyl-2-amino-4-methyl-3-thiophenecarboxylate	227.3	2.47	Yes
SM11	762713	4-[1-(3-methoxyphenyl)cyclopentyl]phenol	268.4	5.01	No
SM12	2849450	3-[3-(4-bromophenyl)-2-triazen-1-yl]-5-nitrobenzoic acid	365.1	4.88	Yes
SM13	6456370	1-benzyl-N-[2-(2,4-dichlorophenyl)ethyl]-4-piperidinamine	363.3	4.85	No
SM14	200186	1-(4-bromophenyl)-3-(dimethylamino)-1-propanone hydrochloride	292.6	2.38	No
SM15	45241167	1-(1-azocanyl)-3-(2-methoxy-5-{[(4-methylbenzyl)amino]methyl}phenoxy)-2-propanol	426.6	4.42	Yes
SM16	5333244	4-bromo-2-{[(3-methoxyphenyl)imino]methyl}phenol	306.2	3.36	Yes
SM17	1909603	1-methyl-5-[(5-nitro-2-furyl)methylene]-2,4,6(1H,3H,5H)-pyrimidinetrione	265.2	1.89	Yes
SM18	97690	4-benzoyl-5-methyl-2-phenyl-2,4-dihydro-3H-pyrazol-3-one	278.3	4.71	Yes
SM19	1548255	1-(9H-fluoren-9-yl)-4-(3-phenyl-2-propen-1-yl)piperazine	366.5	5.94	No

Molecular weight in g/mol; SM: small molecule.

Supplemental Table 2. Bacterial and eukaryotic cell used in this study and their growth conditions

Organisms	Growing conditions	Sources
**Bacterial strains**		
Campylobacter jejuni 81-176	MH broth, 42°C, microaerobic for 2 days	Laboratory collection
Avian pathogenic Escherichia coli O1, O2, O8, O15, O18, O35, O78, O109, and O115 serotypes	M63 minimal broth, 37°C, aerobic for 12 hrs	Laboratory collection
Enterohemorrhagic *Escherichia coli* O157:H7	M63 minimal broth, 37°C, aerobic for 12 hrs	[a]Dr. Jeffrey T. Lejeune
Listeria monocytogenes	LB broth, 37°C, aerobic for 12 hrs	[a]Dr. Jeffrey T. Lejeune
Salmonella Albany*,* Anatum*,* Braenderup*,* Enteritidis, Heidelberg*,* Javiana*,* Muenchen*,* Newport*,* Saint-Paul, Typhimurium	M9 minimal broth, 37°C, aerobic for 12 hrs	Laboratory collection
Salmonella Typhimurium (sulfamethoxazole, streptomycine, and oxyteracycline resistant; n= 3)	M9 minimal broth, 37°C, aerobic for 12 hrs	[c]Dr. John Gunn
Salmonella Typhimurium (streptomycine, ampicillin, and trimethoprim-sulphamethoxazole resistant; n= 1)	M9 minimal broth, 37°C, aerobic for 12 hrs	[c]Dr. John Gunn
Salmonella Typhimurium (ampicillin and sulfamethozaxole resistant; n=1)	M9 minimal broth, 37°C, aerobic for 12 hrs	[c]Dr. John Gunn
Salmonella Typhimurium (ciprofloxacin resistant; n=1)	M9 minimal broth, 37°C, aerobic for 12 hrs	[c]Dr. John Gunn
Salmonella Typhimurium KAN^{R} mutant	M9 minimal broth, 37°C, aerobic for 12 hrs	Laboratory collection
**Eukaryotic organisms**		
Caco-2 cells (ATCC-HTB-37)	MEM + 20% FBS + 1% NEAA + 1 mM sodium pyruvate	[d]ATCC
HD-11 cells (CVCL_4685)	IMEM + 2 mM glutamine + 10% FBS	Dil and Qureshi, 2002
THP-1 cells (ATCC® TIB-202TM)	RPMI 1640 + 10% FBS + 2 mM Glutamine + 100 nM PMA	[d]ATCC
Sheep red blood cells	None	[e]LAMPIRE
Chicken red blood cells	None	OARDC, Ohio
Galleria mellonella (wax moth) fifth instar larva stage	Petri dish + filter paper at 37°C in the dark	Snackworms
One-week-old layer chickens	Metallic cage, at 30°C, lights always on	OARDC, Ohio

[a]Food Animal Health Research Program, OARDC, The Ohio State University, OH, USA; [b]Hørsholm, Denmark; [c]Department of Microbiology, The Ohio State University; [d]American Type Culture Collection, Rockville, MD; [e]LAMPIRE Biological Laboratories,

Pipersville, PA; MRS: Man Rogosa Sharpes; BHI: Brain Heart Infusion; LB: Luria Bertani; MEM: Minimum essential medium; FBS: Fetal Bovine Serum; NEAA: Non-Essential Amino Acids; IMDM: Iscove's Modified Dulbecco's Medium; RPMI: Roswell Park Memorial Institute; PMA: Phorbol 12-Myristate 13-Acetate; KAN^{R}: kanamycin resistant

1. Dil N and Qureshi MA. 2002. Differential expression of inducible nitric oxide synthase is associated with differential Toll-like receptor-4 expression in chicken macrophages from different genetic backgrounds. Vet Immunol Immunopathol 84:191-207.

Supplemental Table 3. SM treatment groups: wax moth study

Groups	Treatment	*S.* Typhimurium*
SM1	12.5 µg	Yes
SM3	12.5 µg	Yes
SM4	12.5 µg	Yes
SM5	12.5 µg	Yes
CK	12.5 µg	Yes
Negative control	30% DMSO	Yes
DMSO control	30% DMSO	No

At the given concentration, SMs were soluble at 30% DMSO and higher. Asterisk: larvae were infected with approximately 3.5 X 10^4 *S.* Typhimurium bacteria per larva 2 hrs after the SM treatment; SM: small molecule; CK: chloramphenicol; n= 20 larva per group

Supplemental Table 4. SM treatment groups: chicken study

Groups	Treatment	*S.* Typhimurium*
SM1	100 µg	Yes
SM3	100 µg	Yes
SM4	100 µg	Yes
SM5	100 µg	Yes
DMSO control	30% DMSO	Yes
Negative control	None	Yes
Positive control	None	No

At the given concentration, SMs were soluble at 30% DMSO and higher. Asterisk: chickens were infected with approximately 10^4 *S.* Typhimurium bacteria per chicken orally 36 hrs prior to the SM treatment; SM: small molecule; n= 10 chickens per group.
