# Supplementary material for: Novel Imidazole and Methoxybenzylamine Growth Inhibitors Affecting Salmonella Cell Envelope Integrity and its Persistence in Chickens
Source: Sci Rep. 2018 Sep 6;8:13381. doi: 10.1038/s41598-018-31249-0 (PMC6127322; doi:10.1038/s41598-018-31249-0)
Supplement: Supplementary file 2 — Supplmental Figures S1-S4 [file 41598_2018_31249_MOESM2_ESM.pdf]

Novel Imidazole and Methoxybenzylamine Growth Inhibitors Affecting *Salmonella* Cell Envelope Integrity and its Persistence in Chickens

Loïc Deblais[1,2], Yosra A. Helmy[1], Dipak Kathayat[1], Huang-chi Huang[1], Sally A. Miller[2], Gireesh Rajashekara[1*].

*Address correspondence to Dr. Gireesh Rajashekara, rajashekara.2@osu.edu.

[1]Food Animal Health Research Program, Department of Veterinary Preventive Medicine, The Ohio State University, OARDC, Wooster, OH.

[2]Department of Plant Pathology, The Ohio State University, OARDC, Wooster, OH.

Small molecules	*Salmonella* serotypes									
	T	Al	A	B	E	H	J	N	SP	M
1										
2										
3										
4										
5										
6										
7										
8										
9										
10										
11										
12										
13										
14										
15										
16										
17										
18										
19										
Hits among the 19 SMs[a]	19	10	8	9	11	6	9	13	10	4
Hits among the 128 SMs[b]	19	13	8	9	12	8	9	18	12	4

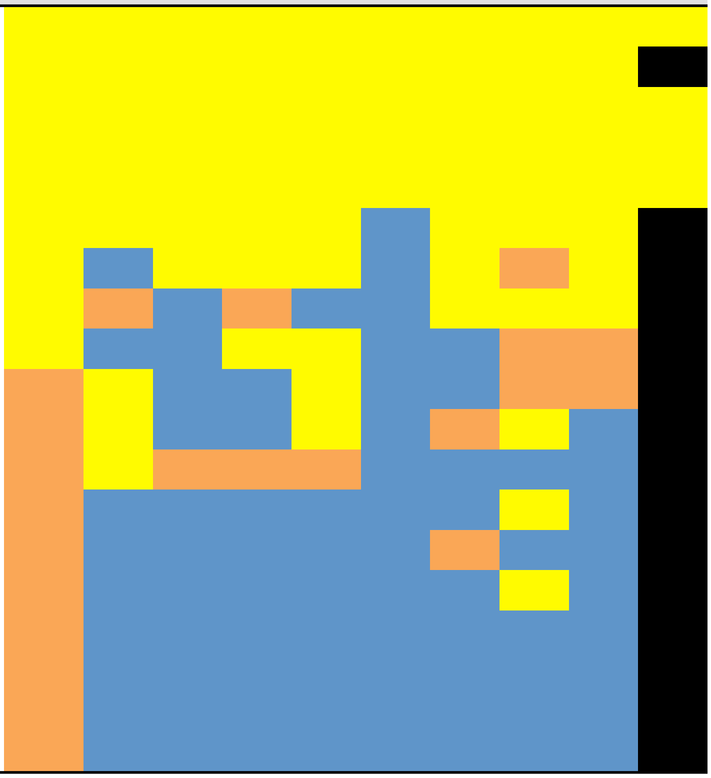

Figure S1. Activity spectrum of the 128 small molecules (SMs) inhibiting at least 20% of *Salmonella enterica* subsp. *enterica* serotype Typhimurium strain JSG626 growth against other *Salmonella* serotypes at 200 µM. Yellow cells: cidal effect; orange cells: static effect; blue cells: bacterial growth observed; black cells: not determined; [a]number of bactericidal and bacteriostatic SMs within the 19 SMs with a bactericidal or bacteriostatic effect against *S.* Typhimurium, for a designated serotype; [b]number of bactericidal and bacteriostatic SMs within the 128 SMs inhibiting *S.* Typhimurium growth (20% or higher), for a designated serotype. T: Typhimurium (JSG626); Al: Albany; A: Anatum; B: Braenderup; E: Enteritidis; H: Heidelberg; J: Javiana; N: Newport; SP: Saint-Paul; M: Muenchen.

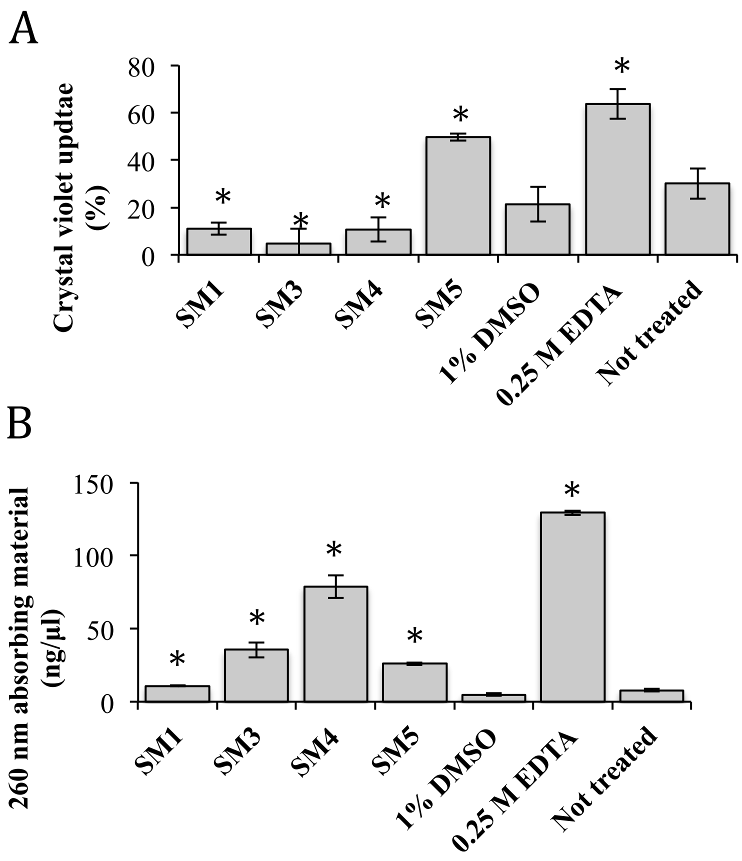

Figure S2. Estimation of the membrane permeability of *Salmonella enterica* subsp. *enterica* serotype Typhimurium strain JSG626 after small molecule (SM) treatment. (A) Crystal violet uptake assay after *S.* Typhimurium was challenged for 1 hr with 5X MBC of SMs. Treatments were normalized based on 10 µg/ml of crystal violet solution (no uptake control). Bars: standard deviation; asterisk: the uptakes of crystal violet was significantly different than the 1% DMSO control (P<0.01); n= 9. (B) Loss of 260 nm absorbing materials from *S.* Typhimurium cell content. 260 nm absorbing materials were measured using a nanodrop once *S.* Typhimurium was challenged for 1 hr with 5X MBC of SMs. Bars: standard deviation; *: the amount of 260 nm absorbing material in supernatant was significantly different than the 1% dimethyl sulfoxide (DMSO) treated cells (P<0.01); n= 9. Both experiments were performed as previously described (1).

1. Devi KP, Nisha SA, Sakthivel R, Pandian SK. 2010. Eugenol (an essential oil of clove) acts as an antibacterial agent against Salmonella typhi by disrupting the cellular membrane. J Ethnopharmacol 130:107–115.

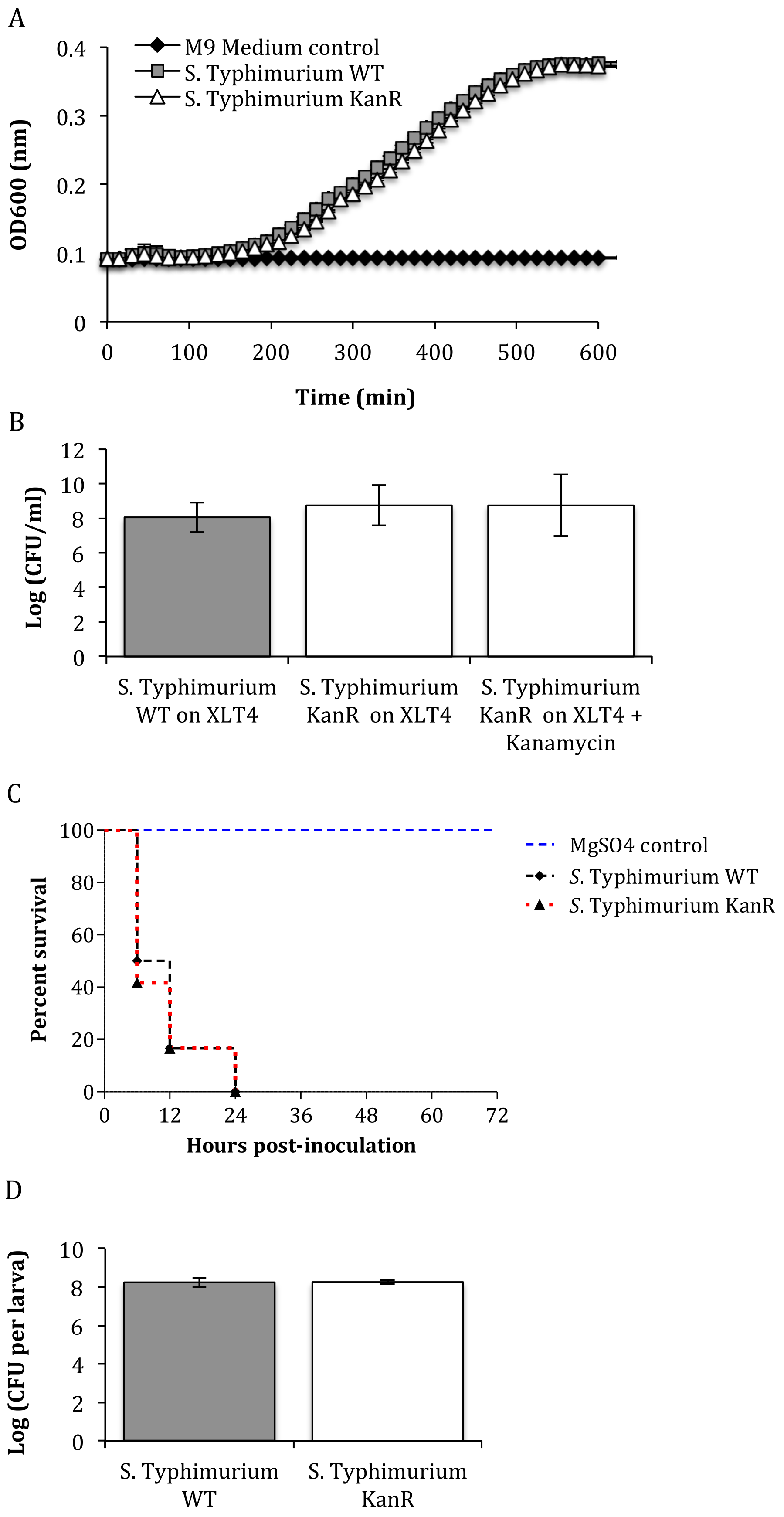
A
M9 Medium control
S. Typhimurium WT
S. Typhimurium KanR
OD600 (nm)
0.4
0.3
0.2
0.1
0
0
100
200
300
400
500
600
Time (min)
B
Log (CFU/ml)
12
10
8
6
4
2
0
S. Typhimurium WT on XLT4
S. Typhimurium KanR on XLT4
S. Typhimurium KanR on XLT4 + Kanamycin
C
MgSO4 control
S. Typhimurium WT
S. Typhimurium KanR
Percent survival
100
80
60
40
20
0
0
12
24
36
48
60
72
Hours post-inoculation
D
Log (CFU per larva)
10
8
6
4
2
0
S. Typhimurium WT
S. Typhimurium KanR

E

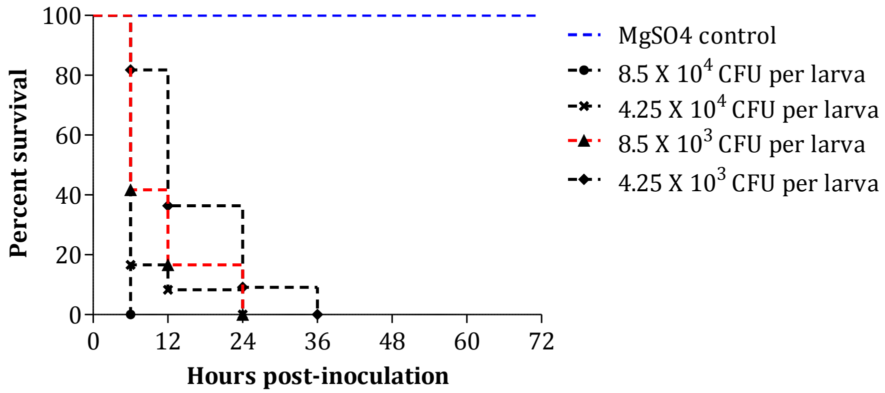
100
80
60
40
20
0
Percent survival
0
12
24
36
48
60
72
Hours post-inoculation
MgSO4 control
8.5 X 10^4 CFU per larva
4.25 X 10^4 CFU per larva
8.5 X 10^3 CFU per larva
4.25 X 10^3 CFU per larva

F

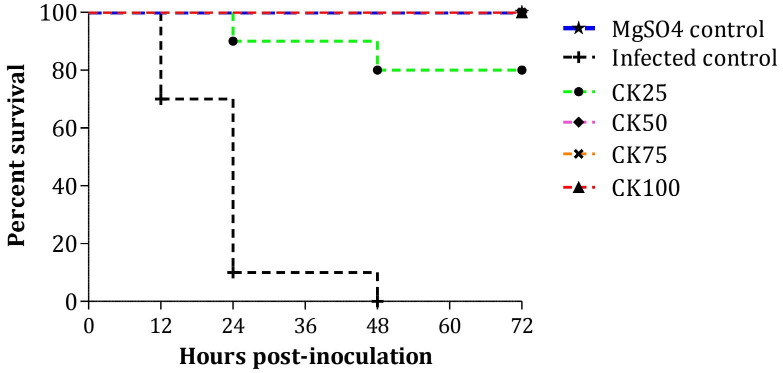
100
80
60
40
20
0
Percent survival
0
12
24
36
48
60
72
Hours post-inoculation
MgSO4 control
Infected control
CK25
CK50
CK75
CK100

Figure S3. A stable chromosomal insertion of pUWGR4 plasmid into *Salmonella enterica* subsp. *enterica* serotype Typhimurium strain JSG626 that did not affect its growth rate and virulence in *Galleria mellonella* larvae. (A) Growth curve of *S.* Typhimurium WT and Kan^R strains for 12 hrs at 37°C in LB broth. Turbidimetric measurement was made every 15 min at 600nm using a Sunrise^{TM} Tecan kinetic microplate reader. N= 4. (B) Bacterial quantification of *S.* Typhimurium WT and Kan^R population after ten growth passages in LB broth (non-selective medium). *S.* Typhimurium WT population was quantified on XLT4 agar while *S.* Typhimurium Kan^R population was quantified on XLT4 agar and XLT4 agar supplemented with 50 µg/ml kanamycin. Bar: standard deviation; n= 4. (C) *G. mellonella* larvae survival rate after infection with either *S.* Typhimurium WT or Kan^R strains. Larvae were infected in the pro-leg with 8.5 X 10^3 CFU per larva. Larvae survival rate was monitored every 12 hrs for three days. Larvae not infected and not treated (NC) or only treated with the MgSO4 buffer (MgSO4) had 100% survival. N= 12. (D) Bacterial quantification of *Salmonella* inside larvae. N= 12; bar: standard deviation. WT: *S*. Typhimurium WT strain; KanR: *S*. Typhimurium Kan^R strain. (E) *G. mellonella* larvae survival rate based on the *Salmonella* inoculum used. Larvae were infected once in a pro-leg with an amount of *S.* Typhimurium Kan^R ranging between 8.5 X 10^2 and 8.5 X 10^4 CFU per larva. Larvae survival rate was monitored every 12 hrs for three days. N= 15. (F) Clearance efficacy of chloramphenicol on *G. mellonella* larvae infected with *S.* Typhimurium. Larvae were treated in a pro-leg 2 hrs before infection with a concentration of chloramphenicol ranging between 25 and 100 mg/kg. Larvae were infected once into another pro-leg with 8.5 X 10^3 *S.* Typhimurium Kan^R per larva. Larvae survival rate was monitored every 12 hrs for three days. Larvae not infected and not treated (NC), treated with the MgSO4 buffer (MgSO4), or treated with 50, 75, and 100 mg/kg of chloramphenicol (CK50, CK75, CK100) had 100% survival. N= 10.

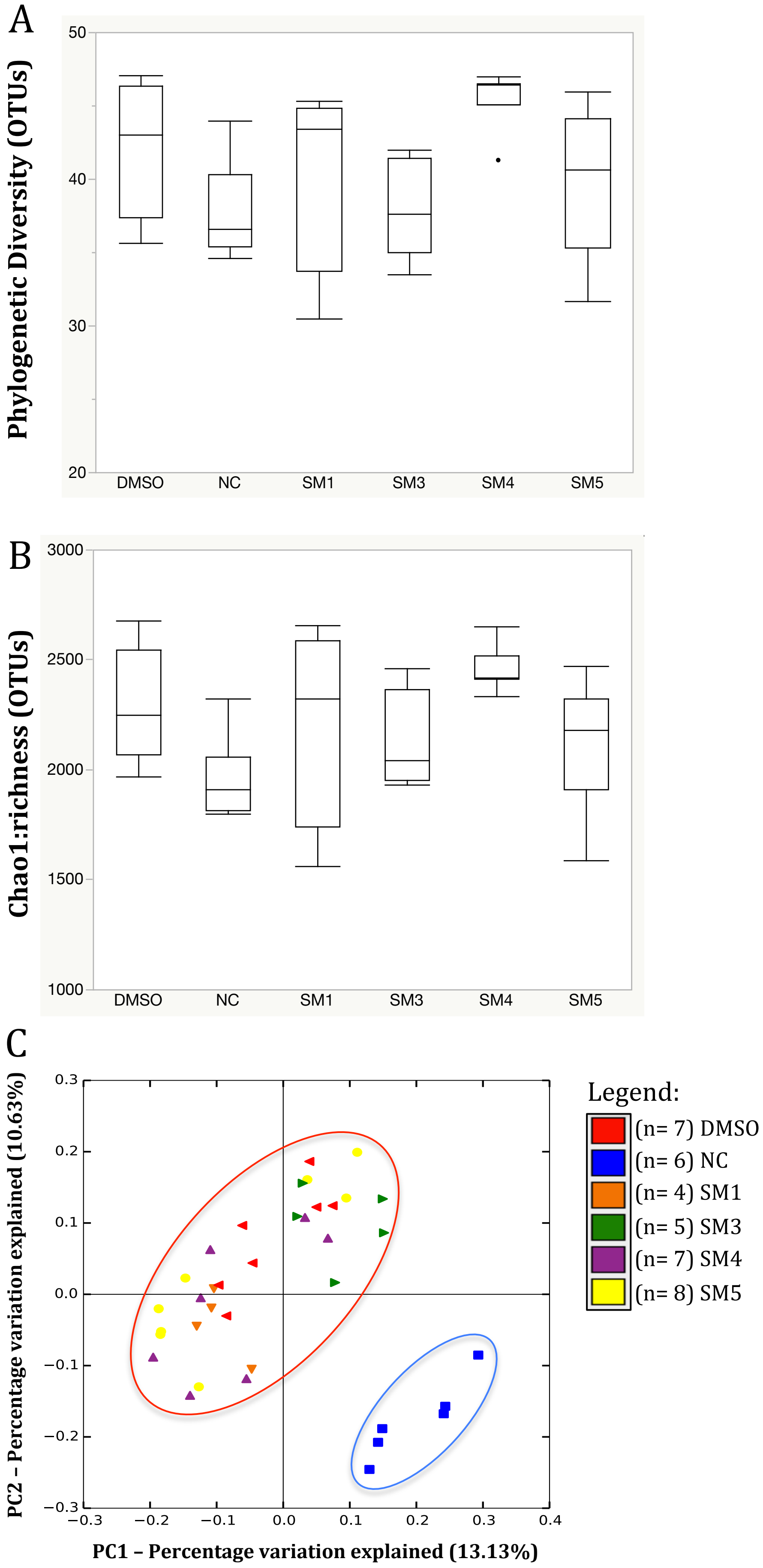
A
Phylogenetic Diversity (OTUs)
50
40
30
20
DMSO
NC
SM1
SM3
SM4
SM5
B
Chao1:richness (OTUs)
3000
2500
2000
1500
1000
DMSO
NC
SM1
SM3
SM4
SM5
C
PC2 – Percentage variation explained (10.63%)
0.3
0.2
0.1
0.0
−0.1
−0.2
−0.3
−0.3
−0.2
−0.1
0.0
0.1
0.2
0.3
0.4
PC1 – Percentage variation explained (13.13%)
Legend:
(n= 7) DMSO
(n= 6) NC
(n= 4) SM1
(n= 5) SM3
(n= 7) SM4
(n= 8) SM5

Figure S4. Impact of small molecule (SM) treatments on the alpha and beta diversities of cecal microbiota in chicken. Alpha diversity was evaluated using the (A) phylogenetic diversity and (B) chao1 (richness). A depth of 14000 sequences was used to study the microbiota samples. (C) Principal coordinate analysis of unweighted uniFrac values. PC: principal coordinate; each dot represent one cecum sample; blue circle: untreated and not infected chicken group (NC); red circle: infected chicken groups treated with DMSO. Additional information concerning the chicken groups are displayed in Table S4.
